# Isolation and Characterization of *Shewanella* Phage Thanatos Infecting and Lysing *Shewanella oneidensis* and Promoting Nascent Biofilm Formation

**DOI:** 10.3389/fmicb.2020.573260

**Published:** 2020-09-18

**Authors:** Maximilian Kreienbaum, Anja K. Dörrich, David Brandt, Nicole E. Schmid, Tabea Leonhard, Fabian Hager, Susanne Brenzinger, Julia Hahn, Timo Glatter, Matthias Ruwe, Ariane Briegel, Jörn Kalinowski, Kai M. Thormann

**Affiliations:** ^1^Department of Microbiology and Molecular Biology, Justus Liebig University Giessen, Giessen, Germany; ^2^Center for Biotechnology, Bielefeld University, Bielefeld, Germany; ^3^Department of Molecular Biotechnology, Institute of Biology, Leiden University, Leiden, Netherlands; ^4^Facility for Mass Spectrometry and Proteomics, Max Planck Institute for Terrestrial Microbiology, Marburg, Germany

**Keywords:** phage, *Shewanella*, biofilm, lysis, adhesion, LPS

## Abstract

Species of the genus *Shewanella* are widespread in nature in various habitats, however, little is known about phages affecting *Shewanella* sp. Here, we report the isolation of phages from diverse freshwater environments that infect and lyse strains of *Shewanella oneidensis* and other *Shewanella* sp. Sequence analysis and microscopic imaging strongly indicate that these phages form a so far unclassified genus, now named *Shewanella* phage Thanatos, which can be positioned within the subfamily of *Tevenvirinae* (*Duplodnaviria*; *Heunggongvirae*; *Uroviricota*; *Caudoviricetes*; *Caudovirales*; *Myoviridae*; *Tevenvirinae*). We characterized one member of this group in more detail using *S. oneidensis* MR-1 as a host. *Shewanella* phage Thanatos-1 possesses a prolate icosahedral capsule of about 110 nm in height and 70 nm in width and a tail of about 95 nm in length. The dsDNA genome exhibits a GC content of about 34.5%, has a size of 160.6 kbp and encodes about 206 proteins (92 with an annotated putative function) and two tRNAs. Out of those 206, MS analyses identified about 155 phage proteins in PEG-precipitated samples of infected cells. Phage attachment likely requires the outer lipopolysaccharide of *S. oneidensis*, narrowing the phage’s host range. Under the applied conditions, about 20 novel phage particles per cell were produced after a latent period of approximately 40 min, which are stable at a pH range from 4 to 12 and resist temperatures up to 55°C for at least 24 h. Addition of Thanatos to *S. oneidensis* results in partial dissolution of established biofilms, however, early exposure of planktonic cells to Thanatos significantly enhances biofilm formation. Taken together, we identified a novel genus of Myophages affecting *S. oneidensis* communities in different ways.

## Introduction

Viruses that prey on prokaryotic organisms, archaea and bacteria, are amazingly abundant in nature. The number of viruses that infect bacteria, which are commonly referred to as bacteriophages or phages, is estimated to surpass that of their host cells by far in most environments (Clokie et al., 2011; Wigington et al., 2016; Parikka et al., 2017). Many phages infect their host cells in a lytic fashion where after infection the cell is instantly directed to produce novel phage particles that are released into the environment by lysis of the host cells. In contrast, lysogenic phages may not immediately kill and lyse their host, but their genome is maintained in the host cell either integrated into that of the host or maintained as independent replicative units. These phages may then proliferate as prophages along with their host cells for many generations before being triggered into the lytic cycle, e.g., by environmental or intracellular signals. In addition, they may exert significant effects on host behavior, fitness and virulence (Argov et al., 2017; Taylor et al., 2019). Thus, phages have a considerable impact on all biological processes on earth from marine environments to the human microbiome (Suttle, 2007; Mirzaei and Maurice, 2017; Breitbart et al., 2018). In addition, recent work, mostly carried out on non-paradigm phage-host pairs, revealed fundamentally novel concepts of phage biology, e.g., that phages use or receive quorum-sensing signals to guide the lysis-lysogeny decisions (Erez et al., 2017; Silpe and Bassler, 2019), that phage group behaviors help to overcome the host defense systems (Landsberger et al., 2018; Nussenzweig and Marraffini, 2018), or that phages may form host-internal structures for proliferation (Chaikeeratisak et al., 2017). The examples illustrate that, although being studied for more than 100 years, many aspects of phages and their interactions with their hosts are not understood and are yet to be discovered.

Species of the genus *Shewanella* are facultatively anaerobic gammaproteobacteria that are most renowned for their ability to use a wide array of respiration pathways to access a plethora of alternative soluble and insoluble electron acceptors (Myers and Nealson, 1988; Hau and Gralnick, 2007; Fredrickson et al., 2008). Therefore, *Shewanella* sp. have some potential to be applied in bioremediation processes or in microbial fuel cell production (Bretschger et al., 2007; Hau and Gralnick, 2007; Logan et al., 2019). Members of this genus occur in a wide range of different environments, such as soils and freshwater and marine sediments (Hau and Gralnick, 2007). In addition, *Shewanella* sp. have been identified in microbiomes of animals and even humans (Tryfinopoulou et al., 2007; King et al., 2012; Dailey et al., 2016; Chen et al., 2017; Flemer et al., 2017), and some species have emerged as human pathogens (Janda and Abbott, 2014; Martin-Rodriguez et al., 2017; Yousfi et al., 2017).

The number of different *Shewanella* species occurring in widely diverse environments strongly implies the existence of an accordingly diverse set of bacteriophages that are able to infect and lyse members of this genus. However, so far relatively few lytic phages for *Shewanella* sp. have been isolated and even fewer of them have been characterized in any detail (Borriss et al., 2003; Han et al., 2014; Luhtanen et al., 2014; Sencilo et al., 2015; Leigh et al., 2017; Wang et al., 2019; Yang et al., 2019). We therefore set out to isolate novel phages using *S. oneidensis* MR-1 as host. *S. oneidensis* is probably the best studied species among *Shewanella*, in particular with respect to its physiology and metabolic and respiratory capabilities (Heidelberg et al., 2002; Beblawy et al., 2018). The genome harbors four prophages, MuSo1, MuSo2, LambdaSo, and CP4So, out of which only MuSo2 and LambdaSo are able to produce infectious particles. However, all prophages have been demonstrated to affect cell physiology and biofilm formation (Qiu et al., 2005; Gödeke et al., 2011; Binnenkade et al., 2014; Zeng et al., 2016; Yao et al., 2018). Here, we isolated two phages, *Shewanella* phages Thanatos-1 and Thanatos-2, which belong to a novel group of phages and are able to infect and lyse several strains of *S. oneidensis* and other *Shewanella* sp. We characterized Thanatos-1 in more detail, and we show that this phage affects biofilm formation of its host in a stage-dependent fashion.

## Materials and Methods

### Bacterial Strains, Growth Conditions, and Strain Constructions

All bacterial strains and plasmids used in this study are listed in Supplementary Table S1. If not indicated otherwise, *Shewanella* strains were routinely grown in liquid LB medium at 30°C, while *Escherichia coli* strains were cultivated in LB medium at 37°C. For solid plates, LB medium was supplemented with 1.5% (w/v) agar if not indicated otherwise. When appropriate, media were supplemented with 50 μg ml^–1^ kanamycin or 10% (w/v) sucrose. Cultures and plates of the *E. coli* conjugation strain WM3064 were always supplemented with 2,6-diamino-pimelic acid (DAP) to a final concentration of 300 μM. All oligonucleotides (Sigma-Aldrich, Taufkirchen, Germany) used for strain constructions are summarized in Supplementary Table S2. Plasmids were constructed using the Gibson assembly method (Gibson et al., 2009). For DNA manipulations and preparations, the appropriate enzymes (Fermentas, St. Leon-Rot, Germany) and kits (VWR International GmbH, Darmstadt, Germany) were used. Plasmids were transferred to *S. oneidensis* via conjugation using *E. coli* WM3065.

### Isolation of *S. oneidensis* Phages Thanatos-1 and -2 and Virion Enrichment

Bacteriophages were isolated from limnetic water and sediment samples incubated with a liquid culture of *S. oneidensis* ΔLambdaSo ΔMuSo2. Following overnight incubation with shaking at 30°C, cells were pelleted by centrifugation and supernatants were filtered through a polyethersulfone (PES) 0.2 μm filter. The presence of target phages in the filtrates was investigated via plaque assays using the double-layer agar method and the strain *S. oneidensis* MR-1 ΔLambdaSo ΔMuSo2. Single plaques were isolated from the plates and again incubated over night with a liquid culture of *S. oneidensis* MR-1 ΔLambdaSo ΔMuSo2 with shaking at 30°C. Cultures were again centrifuged, sterile filtrated and the filtrates were subjected to another round of plaque assays. This singling-out was repeated two times to make sure phage stocks were pure. The number of phages in the final filtrates was assayed using the double-layer agar technique. The undiluted Thanatos-1 phage stock solution used for the experiments contained a phage concentration of ∼10^10^ PFU ml^–1^. Phage preparations were stored at 4°C. For long-term storage, filtrates were supplemented with 10% (v/v) glycerol, frozen in liquid nitrogen and preserved at −80°C.

### Phage Particle Enrichment and DNA Isolation

Phage particles were purified and DNA was subsequently extracted as described earlier (Keen et al., 2017). Usually, 40 ml LB cultures were grown to mid-exponential phase before phages were added and cultures were incubated at RT overnight. In the morning, cells were spun down and, in a new 50 ml tube, 40 ml of the supernatant were supplied with 10 ml of a PEG solution (20% (w/v) PEG 8000, 2.5 M NaCl). The tube was inverted several times before being kept on ice for 1 h. Samples were centrifuged for 75 min at 4000 × *g* and the supernatant was removed. 1 ml STE solution (10 mM Tris, 1 mM EDTA, 100 mM NaCl) was added and used to resuspend the pellet. The suspension was transferred to a 1.5 ml tube and centrifuged for 10 min at 13,000 × *g*. The supernatant containing the phages was kept in a fresh tube at 4°C. If DNA was to be extracted from the purified particles, EDTA and SDS were added to a final concentration of 50 mM and 1%, respectively. Tubes were inverted several times and 250 μl of a phenol:chloroform:isoamyl alcohol mixture (25:24:1) were added. Samples were thoroughly vortexed and subsequently centrifuged at 10,000 × *g* for 4 min. About 700 μl of the supernatant were transferred carefully to a new tube and supplied with 630 μl isopropanol and 70 μl of a 5 M NaCl solution. After gentle inversion, the tubes were again centrifuged at 10,000 × *g* for 10 min. The supernatant was discarded and sediments were washed with 500 μl 70% ethanol by centrifuging for 5 min at 10,000 × *g*. The supernatant was carefully removed and the tubes were left out to dry. Finally, DNA sediments were resuspended in 50 μl of water and stored at 4°C.

### Chromatographic Purification of Phage Particles

For chromatographic purification lysed culture was centrifuged (11,000 × *g*, 10 min, 4°C) and the supernatant was filtered through a 0.2 μm sterile filter. A 1 ml CIMmultus^TM^ OH-1 Advanced Composite Column (Pores 6 μM) was used for chromatography on an ÄKTAprime plus system. Phage lysate was diluted 1:1 with 3 M K_2_HPO_4_, KH_2_PO_4_ buffer (pH 7.0) and loaded on the OH column (flow rate 5 ml/min). Buffer A (1.5 M K_2_HPO_4_, KH_2_PO_4_; pH 7.0) was used for washing and for elution a linear gradient from 0 to 100% of Buffer B (20 mM K_2_HPO_4_, KH_2_PO_4_; pH 7.0) was applied.

### Genome Sequencing, Assembly and Annotation

Sequencing libraries were prepared using the Illumina Nextera library prep kit with 50 ng of input DNA and sequenced on the Illumina MiSeq machine (2 × 300 bp). Genome assembly was performed using Newbler (Margulies et al., 2005) and manually inspected using Consed (Gordon and Green, 2013). Annotation was performed automatically with prokka (Seemann, 2014) and refined with manual annotations from BLASTp homologies using GenDB (Meyer et al., 2003).

### Liquid Chromatography-Mass Spectrometry Analysis on PEG-Precipitated Samples

Samples were acetone precipitated by adding 8 × vol acetone and 1 × vol methanol and incubation at −80°C for 2 h. Protein precipitates were then washed twice with methanol, dried and reconstituted in 300 μl 2% sodium laroyl sarcosinate (SLS). Reduction was carried out for 15 min at 95°C in the presence of 5 mM Tris(2-carboxyethyl)phosphine (TCEP). Alkylation was performed for 30 min at 25°C with 10 mM iodoacetamide. 50 μg total protein was digested overnight at 30°C using trypsin (Promega). Post-digest SLS was precipitated using 1.5% trifluoroacetic acid (TFA) and acidified peptides were used for C18 solid-phase extraction on Microspin columns (Harvard Apparatus) according to manufacturers’ instructions. Purified peptides were dried and reconstituted in 0.1% TFA and applied to the LC-MS system. LC-MS/MS analysis was carried out on a Q-Exactive Plus instrument connected to an Ultimate 3000 RSLC nano and a nanospray flex ion source (all Thermo Fischer Scientific). Peptide separation was performed on a reverse-phase high-performance liquid chromatography column (75 μm × 42 cm) packed in-house with C18 resin (2.4 μm, Dr. Maisch). The peptides were loaded onto a PepMap 100 pre-column (Thermo Fischer Scientific) and eluted by a linear ACN gradient from 2 to 35% solvent B over 60 min (solvent A: 0.15% formic acid; solvent B: 99.85% ACN in 0.15% formic acid). The flow rate was set to 300 nl min^–1^. The peptides were analyzed in positive ion mode. The spray voltage was set to 2.5 kV, and the temperature of the heated capillary was set to 300°C. Survey full-scan MS spectra (*m*/*z* = 375–1500) were acquired in the Orbitrap with a resolution of 70,000 full width at half maximum at a theoretical *m*/*z* 200 after accumulation a maximum of 3 × 10^6^ ions in the Orbitrap. Based on the survey scan, up to 10 most intense ions were subjected to fragmentation using high collision dissociation at 27% normalized collision energy. Fragment spectra were acquired at 17,500 resolution. The ion accumulation time was set to 50 ms for both MS survey and tandem MS (MS/MS) scans. To increase the efficiency of MS/MS attempts, the charged state screening modus was enabled to exclude unassigned and singly charged ions. The dynamic exclusion duration was set to 30 s. Label-free quantification (LFQ) of workflow comparison was performed using Progenesis QI software (Non-linear Dynamics, version 2.0). MS raw files were imported into Progenesis and the output data (MS/MS spectra) were exported in mgf format. MS/MS spectra were then searched using MASCOT against a decoy database of the predicted Thanatos proteome. The following search parameters were used: full tryptic specificity required (cleavage after lysine or arginine residues); two missed cleavages allowed; carbamidomethylation (C) set as a fixed modification; oxidation (M) and deamidation (N,Q) set as a variable modification. The mass tolerance was set to 10 ppm for precursor ions and 0.02 Da for high energy-collision dissociation (HCD) fragment ions. Results from the database search were imported back to Progenesis, mapping peptide identifications to MS1 features. Next, the data obtained from Progenesis was evaluated using SafeQuant R-package version 2.2.2 (Glatter et al., 2012). Hereby, 1% FDR of identification and quantification were calculated including intensity based absolute quantification values (iBAQ; Schwanhausser et al., 2011) and outputs were used for further data extraction.

### LC-MS/MS Analysis of Purified Phage Lysate

For this assay, a ‘single-tube’ preparation protocol was used. For this isolation method (Wang et al., 2005) 100 μl 100 mM ammonium bicarbonate was added to the freeze dried phage elution. The phages were lysed by ten freeze-thaw cycles switching between liquid nitrogen and 37°C. After lysis 100 μl of the organic solvent trifluoroethanol and 5 μl 200 mM of the reducing agent Tris (2-carboxyethyl) phosphine were added and incubated for 60 min at 60°C, followed by the alkylation with 20 μl of 200 mM chloroacetamide for 90 min in darkness. Alkylation was stopped adding 5 μl of 200 mM TCEP and incubate for 60 min at RT. For the tryptic digestion samples were diluted 1:10 with 50 mM ammonium bicarbonate. Digestion takes place at 37°C overnight (Trypsin Gold, Promega). Digested peptides were purified using SepPak columns (Waters, Milford, United States), to secure the nanoLC system. Peptide quantification was done using nanodrop 2000 (Peqlab). Protein was isolated using phenol extraction (Schulte et al., 2017). To this end, subsequent to phage lysis the sample was mixed with water-saturated phenol (Carl Roth, Germany) and incubated on a shaker at room temperature for 30 min, followed by a phase separation via centrifugation. The proteins from the phenolic phase as well as the interphase proteins were precipitated by adding a nine-fold volume of acetone and incubation over night at −20°C. Precipitated proteins were harvested by centrifugation and the resultant sediment was dried at room temperature. Reduction, alkylation and tryptic digestion was done as described earlier (Schelletter et al., 2019). Again, peptides were purified using SepPak columns. The peptides were analyzed using a nanoLC (Ultimate 300, Thermo Fisher Scientific, Germany) coupled to an ESI-Orbitrap MS/MS (QExactive Plus, Thermo Fisher Scientific, Germany). The gradient length of the Acclaim PepMap 100 C18 analytical column was adjusted to 60 min from 4 to 50% of 80% ACN and 0.1% FA. All samples were measured in full MS mode using a resolution of 70.000 (AGC target of 3e6 and 64 ms maximum IT). For the dd-MS^2^ a resolution of 17.500, AGC target of 2e5 and a maximum IT of 100 ms was used. All fractions, “single-tube preparation,” phenol phase and interphase extraction were measured in three technical replicates.

Data analysis was done using MaxQuant (Cox and Mann, 2008; version 1.6.10.43) and Perseus (Tyanova et al., 2016; version 1.6.10.43). Protein databases of Thanatos-1 and *S. oneidensis* and six-frame translation databases of both genomes were used. For protein identification in MaxQuant, digestion enzyme was set to trypsin with a maximal number of two missed cleavages. Standard instrument settings for Orbitrap based data were used as default. All searches include variable modifications of protein N-terminal acetylation and methionine oxidation and carbamidomethylation of cysteines as fixed modifications. The maximal number of modifications per peptide was set to 6. The “second peptide search” was enabled together with the “match between runs” option with the standard parameters. The false discovery rate (FDR) for PSMs and proteins was adjusted to 5%, whereby the FDR for site decoy fraction was used as default. For data interpretation the MaxQuant results were loaded to Perseus software, were all reverse, potential contaminant and “only identified by site” hits were deleted.

### Cryo Electron Microscopy

*S. oneidensis* was grown with shaking at 200 rpm and 30°C in LB to an OD_600__nm_ of 0.3. A volume of 500 μl was harvested and concentrated 10x in 50 μl LB. 5 μl of the respective purified phages was added and the mixture incubated at RT for 2 h. Aliquots of 3 μl were applied to plasma-cleaned R2/2 copper Quantifoil grids (Quantifoil Micro Tools, Jena, Germany) and plunge frozen using a Leica EM GP (Leica microsystems, Wetzlar, Germany) grid plunger. Blotting time was set to 1 s at 20°C and 95% humidity. Grids were stored in liquid nitrogen until imaging. Images were recorded using the Talos L120C (Thermo Scientific) transmission electron microscope equipped with a 4k × 4k Ceta CMOS camera. Acceleration voltage was set to 120 kV and magnification to 36000.

### Phage Spot Assays

The ability of Thanatos to infect different *Shewanella* species, *E. coli* and *Pseudomonas putida* was investigated via phage spot assays. Briefly, 400 μl of cell culture were mixed with 7 ml soft agar [0.5% (w/v)], vortexed vigorously and poured over an LB agar plate. After solidification, 1.5 μl of a 1:10 diluted Thanatos phage suspension (∼10^9^ PFU ml^–1^) were spotted onto each plate with each of the different potential host strains. The formation of plaques was investigated after overnight incubation. To compare the infectivity of Thanatos between different *Shewanella* strains or between different conditions in a semi-quantitative way, the phage suspension (∼10^9^ PFU ml^–1^) was diluted serially in LB medium in a 96-well microtiter plate. Each dilution series was spotted in triplicate onto square shaped double layer agar plates at a volume of 1.5 μl per spot using a multi-channel pipette. For plate preparation, 1.5 ml of a logarithmic phase *Shewanella* cell culture was mixed with 30 ml soft agar [0.5% (w/v)] and poured over a square shaped LB agar plate.

### Lysis Assay During Planktonic Growth

The influence of Thanatos on the growth behavior of *S. oneidensis* was assayed via measuring cell lysis during planktonic growth. Overnight cultures of *S. oneidensis* ΔLambdaSo ΔMuSo2 and wild type were diluted in fresh LB medium to an OD_600__nm_ of ∼0.1 and incubated at 30°C with shaking for 2 h. When the cell cultures reached an OD_600__nm_ of ∼0.4, they were infected with Thanatos phage at a multiplicity of infection (MOI) of 0.1. Samples were taken every hour and the OD_600__nm_ was plotted against time.

### Temperature and pH Sensitivity Assays

To investigate the thermal resistance of Thanatos, undiluted phage preparations (∼10^9^ PFU ml^–1^) were incubated at room temperature (RT), 50°C, 55°C, 60°C and 65°C in a thermo-block (Eppendorf) for 24 h. To assay pH stability, 10 μl of a phage preparation (∼10^9^ PFU ml^–1^) were incubated for 24 h with 90 μl of LB medium adjusted to different pH values at RT. Following incubation under the indicated conditions, dilution series were created using LB medium and 1.5 μl of each dilution were spotted onto square shaped double-layer agar plates containing *S. oneidensis* ΔLambdaSo ΔMuSo2.

### Latent Period and Phage Burst Size

To determine the latent period and burst size, a one-step growth experiment was performed as described earlier (Adams, 1959; Chow et al., 1988). Briefly, 50 ml of cell culture (*S. oneidensis* ΔLambdaSo ΔMuSo2) was incubated to an OD_600__nm_ ∼0.4 and harvested by centrifugation. The pellet was resuspended in 0.5 ml of LB medium and mixed with 10 μl of Thanatos phage solution (∼10^10^ PFU ml^–1^). The phages were allowed to adsorb for 1 min. After adsorption, free phage particles were removed by centrifugation. The cell pellet was resuspended in 100 ml of fresh LB medium and the culture was subsequently incubated at 30°C with shaking. At 5 min intervals, 250 μl samples were taken, mixed with 5 ml of soft agar [0.3% (w/v)] and poured over an LB plate. Resulting plaques were quantified following over-night incubation at 30°C. The burst size was calculated by dividing the average phage titer at the plateau phase by the average phage titer along the latent phase. Duration of the latency period was verified by investigating cell lysis of an infected culture via light microscopy.

### Light Microscopy of Infected *Shewanella* Cells

*S. oneidensis* ΔLambdaSo ΔMuSo2 was cultured overnight in LB medium and subcultured the next day to exponential growth phase (OD_600__nm_ ∼0.3–0.6). The cells in a culture aliquot of 200 μl were harvested, washed twice with PBS buffer, infected with 200 μl Thanatos phage solution (∼10^10^ PFU ml^–1^) and incubated at RT for 5 min. Afterward, 3 μl of culture were spotted on an agarose pad [1% (w/v) agarose in LM medium (10 mM HEPES, pH 7.5; 100 mM NaCl; 0.02% yeast extract; 0.01% peptone; 15 mM lactate)]. Images were recorded in 5–10 min intervals using a Leica DMI 6000 B inverse microscope (Leica, Wetzlar, Germany) equipped with an sCMOS camera and a HCX PL APO 100×/1.4 objective using the VisiView software (Visitron Systems, Puchheim, Germany). Image processing was carried out using the Fiji tool (Schindelin et al., 2012).

### Transposon Mutagenesis

Transposon mutagenesis was performed in order to determine the receptor used by Thanatos for adsorption to its host. The plasmid pMiniHimar RB1 (Bouhenni et al., 2005) was transferred into *S. oneidensis* ΔLambdaSo ΔMuSo2 from *E. coli* WM3064 by conjugation. Single clones were streaked on LB agar plates supplemented with kanamycin (50 μg ml^–1^) and on plates containing kanamycin, on which in addition 10^9^ PFU of phages had been plated. Mutants resistant to kanamycin and to Thanatos infection were picked and again transferred to two plates, one containing kanamycin (50 μg ml^–1^) and one containing kanamycin on which virulent Thanatos phage had been spread homogeneously at 10^9^ PFU per plate. Colonies with a uniform, round shape on both plates were selected as phage resistant mutant candidates. To exclude false positives, these candidates were screened in a phage spot assay. Clones resistant to Thanatos infection (no phage plaque emerged) were restreaked on LB agar plates containing 50 μg ml^–1^ kanamycin. From these candidates, chromosomal DNA was isolated using the E.Z.N.A. Bacterial DNA Kit (Omega Bio-tek, Norcross, United States). To create plasposons, the DNA was digested with *Bam*HI (Thermo Fisher Scientific, United States) and the fragments re-ligated with T4 DNA ligase (Thermo Fisher Scientific, United States). *E. coli* DH5αλ*pir* was transformed with the plasposons. Plasposon DNA from clones being able to grow on kanamycin (50 μg ml^–1^) was isolated using the E.Z.N.A. Plasmid Mini Kit I (Omega Bio-tek, Norcross, United States.) and sequenced at Microsynth Seqlab (Göttingen, Germany) using Primers FR24 and FR25. Sequence data were used to identify the site of transposon insertion.

### Phage Adsorption Assay

The phage adsorption assay was performed to verify that the deletion of *waaC* prevents the adsorption step itself rather than a different step of the bacteriophage life cycle. The strains to be investigated (*S. oneidensis* ΔLambdaSo ΔMuSo2 and ΔLambdaSo ΔMuSo2 Δ*waaC*) were incubated in LB medium until they reached mid logarithmic phase. These cultures were used to prepare 10 ml main cultures with an exact OD_600__nm_ of 0.15. At time point zero (t0), 1 ml of phage lysate (2 × 10^6^ PFU ml^–1^) was added to the cultures. After 3, 6 and 9 min post infection, 50 μl samples were taken and mixed with 950 μl ice cold LB medium, which had previously been supplemented with 20 μl chloroform. As a control, 1 ml of phage lysate was added to 10 ml LB medium in order to determine the phage concentration at t0. Afterward, the PFU per ml in the samples were quantified. Between 10 and 100 μl of the samples were mixed with 400 μl host cell culture (*S. oneidensis* ΔLambdaSo ΔMuSo2) and 7 ml soft agar [0.5% (w/v)], vortexed vigorously and poured over an LB agar plate. Plaques were counted following over-night incubation at RT.

### Static Biofilm Assays

The influence of Thanatos phage on the biofilm formation of *S. oneidensis* and *S. oneidensis* ΔLambdaSo ΔMuSo2 was investigated by performing static biofilm assays. In brief, a 96-well microtiter plate was inoculated with 200 μl *S. oneidensis* cell culture per well diluted to an OD_600_ of 0.15 in LB medium and incubated 24 h at 30°C. For short-term assays, Thanatos phage (∼10^6^ PFU in 10 μl LB medium) was added to the wells 0, 3, 6 and 22 h post inoculation, during the 24 h incubation period. In this way, the impact of Thanatos on the developing biofilm could be investigated. As a control, the assay was performed with Thanatos particles that were heat-inactivated at 75°C for 60 min. For long-term assays, the inoculated 96-well plate was incubated without the addition of phages for 24 h at 30°C. After 24 h, Thanatos phage (∼10^6^ PFU in 10 μl LB medium) was added to the mature biofilm using the same concentration as above and incubated for further 2, 6, 9 or 24 h, respectively. Prior to staining, the OD_600_ was determined to measure bacterial growth. Surface-associated biomass was stained with 12 μl 0.5% (w/v) crystal violet per well for 10 min at room temperature. The supernatant was removed, the wells were washed with 200 μl distilled water and 200 μl 96% (v/v) ethanol was added to redissolve the retained crystal violet. Surface-associated biomass was quantified by measuring the absorbance at 580 nm and calculated as the percentage of treated cultures (addition of infectious Thanatos or heat-inactivated particles, respectively) compared to untreated cultures (addition of an equal amount of LB medium). All assays were repeated three independent times. For creation of the box and whisker plots, outliers according to Tukey were omitted from the graph.

### Static Biofilm Staining and Fluorescent Imaging

To determine the amount of eDNA in biofilms, *S. oneidensis* and *S. oneidensis* ΔLambdaSo ΔMuSo2 were cultivated in 300 μl volume under static biofilm conditions (described above) in an uncoated 8 well μ-slide (Ibidi, Germany). Then, ∼1.5 × 10^6^ PFU in 10 μl Thanatos phage particles were added into the wells at the appropriate time point post inoculation. After 24 h of incubation at 30°C, the medium containing non-attached biomass was carefully removed and biofilms were carefully washed with 1 × PBS (137 mM NaCl; 2.7 mM KCl; 10 mM Na_2_HPO_4_; 1.8 mM KH_2_PO_4_). Staining of vital cells was performed using 5 μM SYTO 9 (Molecular Probes; Invitrogen, United States.), whereas staining of dead cells and eDNA was performed with 30 μM propidium iodide (Thermo Fisher Scientific, United States) in 300 μl 1 × PBS for 10 min in the dark. After staining, cells were carefully washed once in 1 × PBS and remained in 1 × PBS for microscopic analysis. Imaging was performed using the set-up described above for light microscopy using the APO ×63/1.4–0.6 objective (Leica, Germany).

### Accession Numbers

The sequences of Thanatos-1 and Thanatos-2 are deposited under GenBank accessions MT457552 and MT457553.

## Results

### Isolation of Novel Phages Infecting and Lysing *S. oneidensis*

**FIGURE 1:**
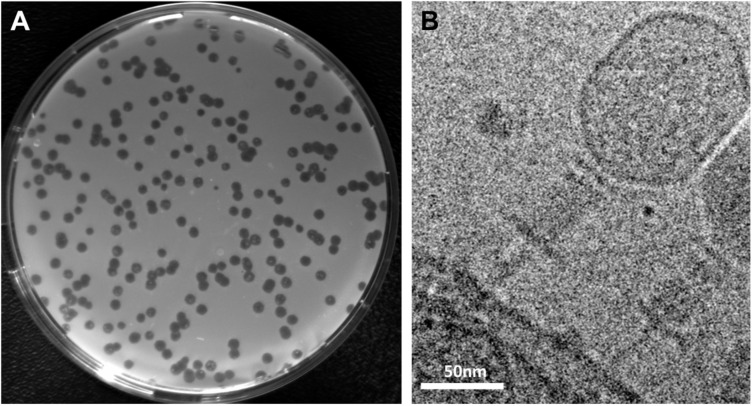
Plaque formation and morphology of *Shewanella* phage Thanatos. **(A)** Plaques formed by phage Thanatos-1 on agar overlay plates using *S. oneidensis* ΔLambdaSo ΔMuSo2 as host strain. **(B)** Cryo electron micrograph of phage Thanatos-1 visualized in a lysed culture of *S. oneidensis* ΔLambdaSo ΔMuSo2. All isolates showed a highly similar plaque morphology and appearance.

*S. oneidensis* was originally isolated from a freshwater sediment (Myers and Nealson, 1988). To identify novel phages infecting this species, we therefore sampled sediments of small streams, ditches and ponds and performed phage enrichment followed by plaque assays. To avoid a potential interference with the active lysogenic phages already present in *S. oneidensis*, we used a strain in which LambdaSo and MuSo2 were completely deleted (ΔLambdaSo ΔMuSo2). From four sediment samples each from a different location, phages forming distinct clear plaques (Figure 1A) were obtained. The phages were directly isolated from plaques on the plate and passaged three times from a single plaque prior to further characterization.

To determine the phage morphology, phage particles were imaged by cryo electron microscopy (Figure 1B). The virions of all isolates displayed a highly similar appearance with a prolate icosahedral head, and a tail with a collar structure. The head had a length of about 110 nm and a width of about 70 nm and is thus somewhat smaller than that of the T4 phage (115 nm/85 nm; Yap and Rossmann, 2014). The tails exhibited an overall length of about 95 nm. However, it appeared that these tails consisted of a contracted sheath of about 45 nm in length and about 22 nm in width, indicated by the presence of a potential baseplate, which is decorated by tail fibers. The part beneath the putative base plate resembles an inner tube extending from the sheath. This general morphology strongly suggested that the newly isolated phages belong to the family of *Myoviridae* within the order *Caudovirales* (Ackermann, 2009), which possess a contractive tail. However, under the conditions tested, all phage tails similarly exhibited this appearance and length, which may suggest that all phage particles that were imaged under these conditions had an already contracted tail.

### Classification of the Novel *Shewanella* Phages

For further characterization and classification, we performed sequencing of the phages genomes. All four phage isolates were found to contain a linear dsDNA chromosome. The genomes of three isolates were identical at the sequence level, thus, two different *S. oneidensis* phages were identified. The two novel isolates were named *Shewanella* phage Thanatos-1 and Thanatos-2, which have genome sizes of 160,584 bp (34.5% G+C) and 155,580 bp (34.8% G+C), respectively. The G + C content is thus considerably lower than that of the host bacterium *S. oneidensis* (45.9%). Thanatos-1 and Thanatos-2 exhibit strong homology at the DNA sequence level (87.8%) and gene synteny (Supplementary Figure S1), indicating that both isolates are closely related. Genome analysis suggests the presence of 206 and 193 protein-encoding open reading frames, respectively, as well as two tRNAs, (Arg-TCT and Met-CAT) in each phage genome. Thanatos-2 has two predicted coding sequences annotated as large terminase subunit, which are caused by a 1.2 kbp insertion in the coding sequence leading to a frameshift. Remaining differences between both phages are mostly hypothetical proteins or proteins with unknown functions. The sequences of Thanatos-1 and Thanatos-2 are deposited under GenBank accessions MT457552 and MT457553.

**FIGURE 2:**
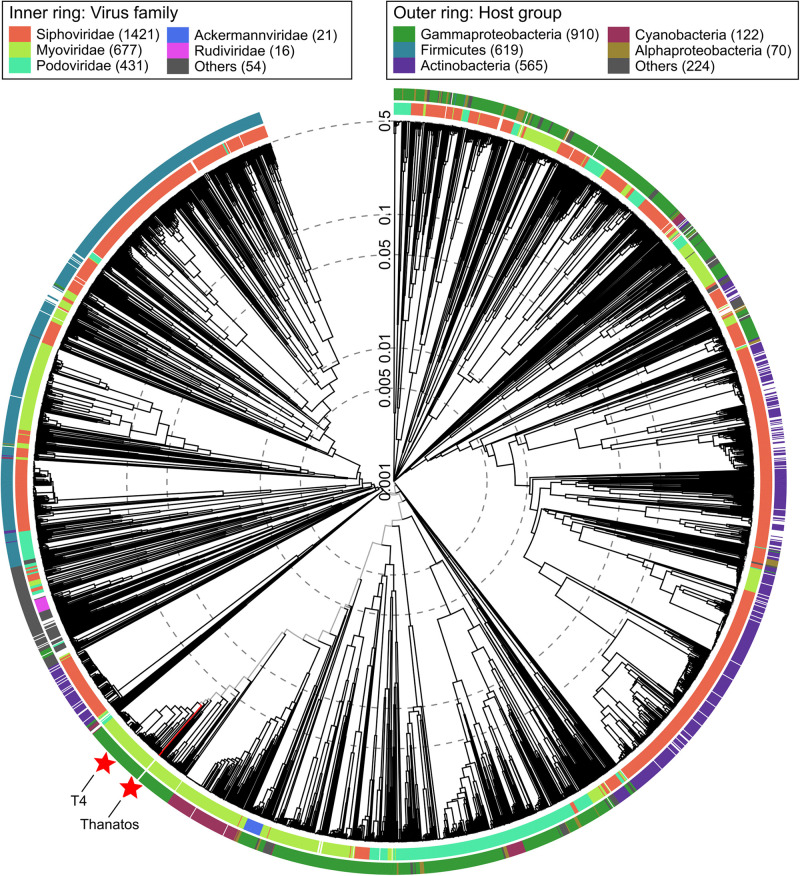
Viral proteomic tree. Phylogenetic tree created using ViPTree (Nishimura et al., 2017) (available at: https://www.genome.jp/viptree/). The proteomic tree is generated based on genome-wide similarities as determined by tBLASTx. Thanatos phages and all genome sequences of phages from the order *Caudovirales* from Virus-Host DB (RefSeq release 93) were included in the analysis (ref). Outer and inner ring are colored according to host group and virus family, respectively. For reference, Thanatos phages and *Escherichia* virus T4 are highlighted by red stars.

A first phylogenetic classification by viral proteomic tree analysis placed both phages in the order *Myoviridae* and subfamily *Tevenvirinae* outside of any existing genus (Figure 2 and Supplementary Figure S2) (Nishimura et al., 2017). During phage genome annotation, multiple protein sequences from *Pseudomonas* phage PspYZU05 (GenBank: KY971610; unpublished) returned the best BLASTp hits. Accordingly, PspYZU05 was included in further phylogenetic analyses, which were also conducted based on multiple sequence alignments of the large terminase subunit and DNA polymerase protein sequences using Clustal Omega (Sievers et al., 2011). All phages from *Myoviridae* subfamily *Tevenvirinae* were included in the single gene-based phylogeny analyses as well. After multiple sequence alignment, Maximum-Likelihood consensus trees were calculated using IQ-Tree (Trifinopoulos et al., 2016). Thanatos-1 and Thanatos-2 were placed in direct vicinity of several unclassified *Acinetobacter* phages and the Enterobacteria phage genus *Krischvirus* (Supplementary Figures S3, S4) according to the large terminase subunit protein sequence and next to the genus *Gaprivervirus* based on the DNA polymerase genus, respectively. Furthermore, the viral proteomic tree was regenerated including PspYZU05 and confirmed the large terminase subunit- and DNA polymerase-based phylogenetic classifications (not shown). The findings suggest that *Shewanella* phages Thanatos-1 and Thanatos-2 belong to a new genus inside the subfamily *Tevenvirinae*. For further characterization, we focused on *Shewanella* phage Thanatos-1 (in the following simply referred to as Thanatos).

**FIGURE 3:**
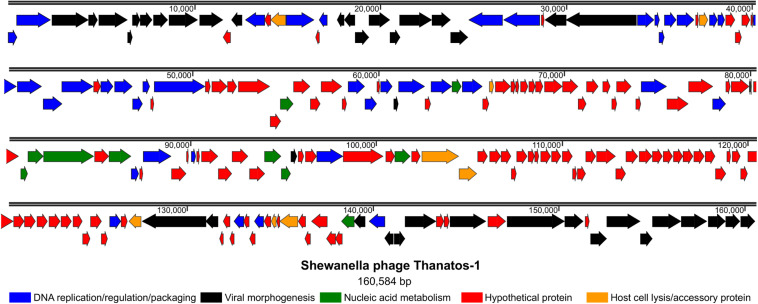
Genome organization of *Shewanella* phage Thanatos. The image displays the predicted open reading frames within the phage genome indicating their transcriptional direction. The deduced gene products are color-coded as indicated below according to their predicted functional categories. The ORF plot has been generated using SnapGene software (from Insightful Science; available at snapgene.com). For the corresponding annotations see Supplementary Table S3.

Among the 206 open readings frames that were identified within the chromosome of phage Thanatos, a putative function could be deduced for only 92 gene products according to amino-acid sequence similarities to known proteins (Figure 3 and Supplementary Table S3). These include proteins required for (i) DNA replication, regulation, and packaging, (ii) viral morphogenesis, (iii) nucleic acid metabolism as well as (iv) host cell lysis and accessory proteins. The gene arrangement indicates a modular organization of functional groups across the phage genome. Thanatos possesses 66 genes with homologs in well-described *Escherichia* virus T4 according to BLASTp analysis. Homologous genes comprise those encoding essential structural proteins as well as proteins functionally involved in DNA replication and packaging (Figure 3), suggesting that several of Thanatos’ main functions may be similar to those of T4 and several of the genes display a similar clustering as in the T4 genome (see Supplementary Figure S5).

To identify the proteins present in the infectious phage particles, we performed mass spectrometry (MS) analysis on purified Thanatos virions. This analysis identified 104 proteins, including all potential main Thanatos structural subunits (Supplementary Table S3). However, some high-abundance *S. oneidensis* proteins were also identified, suggesting that the sample still contained some protein contaminations. A broader MS analysis on PEG-precipitated Thanatos-infected cultures of *S. oneidensis* reliably identified 155 phage proteins (Supplementary Table S3). Both preparations revealed the presence of a number of those annotated as hypothetical proteins. This applied in particular to the PEG-precipitated culture sample, strongly suggesting an important but yet undefined role of these unknown proteins in propagation of phage Thanatos in *S. oneidensis*.

### Propagation and Stability of *Shewanella* Phage Thanatos

**FIGURE 4:**
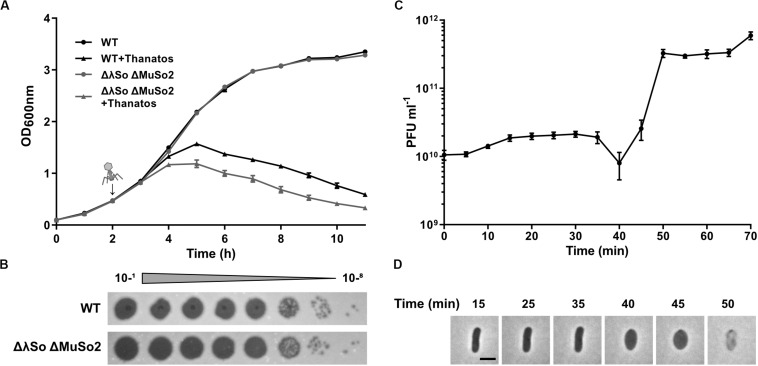
Infection dynamics, latent phase and burst size of Thanatos. **(A)** Lysis assay during planktonic growth of *S. oneidensis* WT and ΔLambdaSo ΔMuSo2 infected with Thanatos compared to uninfected cultures. Phages were added 2 h post inoculation (represented by the arrow) at an MOI of 0.1. The experiment was performed in three biological replicates with technical duplicates each. Error bars represent the SEM. **(B)** Phage spot assay of Thanatos on *S. oneidensis* WT and ΔLambdaSo ΔMuSo2. The 10^– 1^ dilution equals a phage concentration of ∼10^9^ PFU ml^– 1^. **(C)** One step growth experiment. *S. oneidensis* ΔLambdaSo ΔMuSo2 was chosen as host strain. The burst size was calculated by dividing the average phage titer at the plateau phase by the average phage titer along the latent phase. Error bars represent the SEM. **(D)** Time lapse analysis of a *S. oneidensis* ΔLambdaSo ΔMuSo2 cell infected with Thanatos. The scale bar equals 2 μm.

**FIGURE 5:**
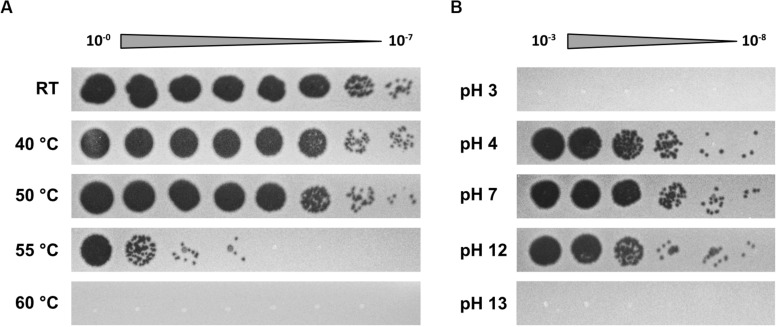
Temperature and pH sensitivity of Thanatos. **(A)** Thermal stability test (24 h). **(B)** pH stability test (24 h). The undiluted phage preparation contained ∼10^9^ PFU ml^– 1^. *Shewanella oneidensis* ΔLambdaSo ΔMuSo2 served as host strain. All images are representatives from three biological replicates.

To further determine phage propagation characteristics, exponentially growing cultures of *S. oneidensis* ΔLambdaSo ΔMuSo2 at an OD_600_ 0.5 were infected with Thanatos at an MOI of 0.1 (Figure 4A). Growth of the infected culture decreased significantly after 1 h of further incubation before the OD_600_ dropped to very low levels after about 12 h, strongly indicating massive lysis of the host culture. The same behavior was observed for cultures of wild-type *S. oneidensis* containing the lysogenic phages LambdoSo and MuSo2, possibly with a slight delay in the onset of lysis, but producing the same amount of phage particles (Figure 4B). Thus, the presence of the prophages does not exert a pronounced positive or negative effect on Thanatos infection. To further determine phage production parameters, we performed a one-step growth experiment under the same conditions at room temperature and in complex media (Figure 4C). Under these conditions, Thanatos exhibited a latent period of about 40–45 min followed by a burst phase of 5–10 min. The number of infectious phage particles released per lysed cell was about 20. Infection experiments were also carried out using time-lapse microscopy on immobilized cells. Infected cells did not show any indications of growth or initiating cell division but rounded up after 40–45 min to finally lyse (Figure 4D), thus well matching the results from the liquid culture. The released phage particles remained at least partly infectious at temperatures of up to 55°C for 24 h before fully losing activity at 60°C (Figure 5A). Almost full infectivity was observed in a pH range from 4 to 12 (for 24 h) (Figure 5B).

### Thanatos Host Recognition Requires the *waaC* Gene of *S. oneidensis*

We then tested the host range of phage Thanatos. To this end, we performed plaque assays on a set of 28 *Shewanella* sp. (see Supplementary Table S4), among them a set of wild-type *S. oneidensis* isolates (Jung-Schroers et al., 2018) as well as an *S. putrefaciens* CN-32 strain with or without its CRISPR-Cas system (Dwarakanath et al., 2015). In addition to *S. oneidensis* MR-1, lysis occurred with several, but not all *S. oneidensis* isolates (four out of eight) as well as with two (out of five) *S. seohaensis* isolates and *Shewanella* sp. MR-7. All other isolates as well as *E. coli* MG1655 and *Pseudomonas putida* KT2440 were not susceptible to *Shewanella* phage Thanatos, which therefore appears to be rather strain- than species-specific for *Shewanella*.

**FIGURE 6:**
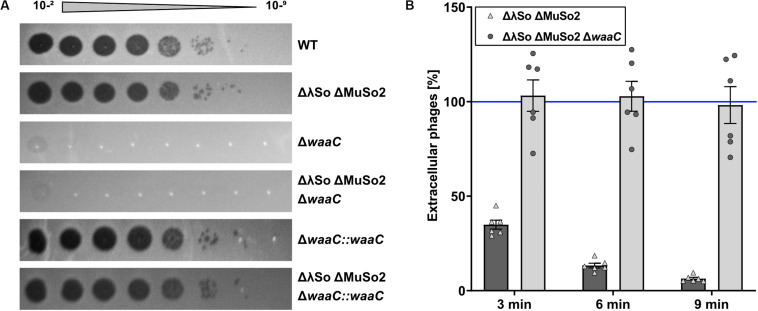
Deletion of *waaC* protects *Shewanella oneidensis* from Thanatos infection by preventing phage adsorption. **(A)** Spot Assay of Thanatos on *Shewanella oneidensis* WT, ΔLambdaSo ΔMuSo2, *waaC* deletion and *waaC* complementation strains. The 10^– 2^ dilution equals a phage concentration of ∼10^8^ PFU ml^– 1^. **(B)** Phage adsorption assay. The cells were grown to early exponential phase (OD_600_ 0.15), before 2 × 10^6^ PFU ml^– 1^ were added to the culture. Samples were taken at the indicated time points and the number of PFU was determined.

To identify potential surface structures that Thanatos uses to recognize its host, we determined the infectivity of Thanatos toward strains of *S. oneidensis* bearing mutations in different surface structures such as flagella (Δ*flaAB*, Δ*flgE*, Δ*pomAB*), type IV pili and type I fimbriae (Δ*pilMQ*, Δ*csgAB*, Δ*csgEFG*, Δ*gspD*), or extracellular polysaccharides (Δ*mxdABCD*). None of those mutants became resistant toward phage infection (data not shown). We therefore performed a random transposon mutagenesis approach and screened for mutants resistant to phage infection on plates and in liquid media. Two independent mutants were isolated from this screening, and in both mutants the site of transposon integration was mapped to the gene SO_4678, annotated as *waaC* encoding an ADP-heptose-LPS heptosyltransferase. In *E. coli*, WaaC is involved in formation of the outer membrane lipopolysaccharide (Grizot et al., 2006), suggesting that phage Thanatos recognizes the LPS of *S. oneidensis*. To validate this finding and to rule out potential polar effects by the transposon insertion, an in-frame deletion mutant of *waaC* was constructed in the wild type and the ΔLambdaSo ΔMuSo2 mutant of *S. oneidensis*. Loss of *waaC* rendered both strains resistant to Thanatos infection (Figure 6A), and this phenotype was fully complemented upon reinsertion of *waaC* into its native genome locus. Also Thanatos-2 was unable to infect *S. oneidensis waaC* mutants (Supplementary Figure S6), strongly indicating that both phages recognize a WaaC-dependent receptor.

As a second line of evidence, we conducted a phage adsorption assay (Figure 6B). To this end, Thanatos was added to *S. oneidensis* MR-1 ΔLambdaSO ΔMuSO2 cells at an MOI of 0.1 and the amount of remaining phage particles in the supernatant was determined over time. Under the conditions tested, Thanatos phages almost completely attached to their host cells after 6–9 min. In contrast, the phage particles were unable to bind to cells in which *waaC* was deleted. Taken together, these results show that WaaC is strictly required for attachment and infection of *S. oneidensis* by phage Thanatos.

### Presence of Thanatos Differentially Affects *S. oneidensis* Biofilms

**FIGURE 7:**
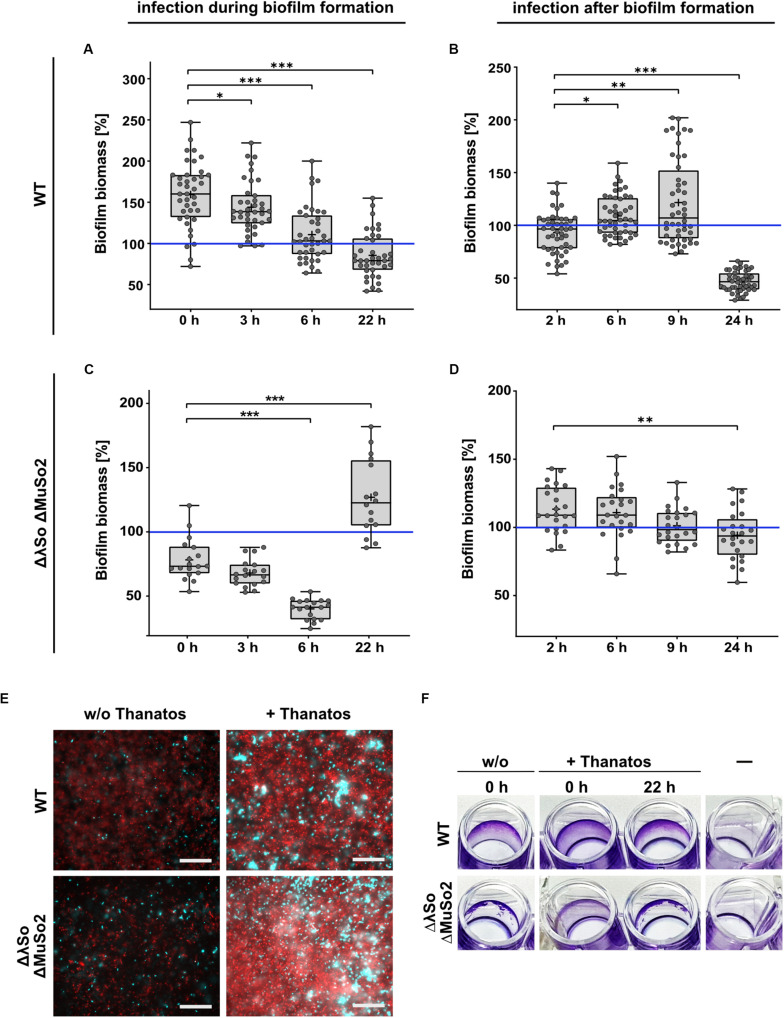
Thanatos differently influences developing and mature biofilms depending on the presence of prophages. Shown is the biofilm formation under static conditions of infected *Shewanella oneidensis* WT and *Shewanella oneidensis* ΔLambdaSo ΔMuSo2 cultures compared to uninfected cultures. Values of panels **(A–D)** represent the percentage of biofilm biomass of treated cultures in comparison to untreated cultures. Biofilm biomass of the untreated cultures was set to 100% (indicated by the blue line). **(A,C)** Short term biofilm assay. Cultures were inoculated in 96 well microtiter plates at 0 h. The time points indicate the addition of phages to the developing biofilm. Absorption of crystal violet was measured after 24 h. **(B,D)** Long-term biofilm assay. After inoculation of the cultures, the microtiter plate was incubated without the addition of phages. After 24 h, infectious phages were added to the mature biofilms. Absorption of crystal violet was measured 2, 6, 9 and 24 h after the addition of phages. *p < 0.05; **p < 0.01; ***p < 0.0001. **(E)** Microscopic analysis of biofilms. 24 h-old biofilms were counterstained with SYTO9 (cyan) and propidium iodide (red). Scale bars = 50 μm. **(F)** 96-well microtiter plate showing the macroscopic biofilm morphology. A static biofilm assay was performed and the biofilms were documented after removal of crystal violet.

Previous studies showed that phages can have a significant impact on *Shewanella* biofilms (Gödeke et al., 2011; Binnenkade et al., 2014; Leigh et al., 2017). We therefore determined if exposure to Thanatos has any effect on the formation or the maintenance of *S. oneidensis* communities. To characterize a potential influence on developing biofilms, we used the *S. oneidensis* wild type as well as the mutant lacking the prophages LambdaSo and MuSo2, as the latter displays a severe biofilm phenotype (Gödeke et al., 2011; Binnenkade et al., 2014). Both *S. oneidensis* strains were cultivated under static conditions in microtiter plates and a defined amount of Thanatos particles were added immediately together with the cells (*t* = 0) or 3, 6, or 22 h after the cells. After 24 h, the amount of planktonic cells and surface-associated biomass was determined. The values were compared to cultures to which no Thanatos particles were added. As a control, the same assay was carried out in a parallel approach using heat-inactivated Thanatos. We found that early addition of active Thanatos particles to wild-type cells enhanced the amount of surface-associated biomass by more than 50% on average (Figure 7A). This enhancing effect became gradually smaller with prolonged pre-incubation and was almost absent when Thanatos was added to 6 h-old biofilms. In contrast, after immediate addition of Thanatos to *S. oneidensis* mutant cells lacking the active prophages, the overall amount of surface-associated biomass was slightly lower than that of untreated cells (Figure 7C). This effect was enhanced upon later Thanatos addition after 3 and 6 h. However, we noticed a change of the biofilm morphology (Figure 7F): Untreated mutant cells almost exclusively form a ring within the microtiter well close to the liquid-air interface, while the remaining surface was almost free of cells. In contrast, Thanatos-exposed mutant cells displayed a morphology rather resembling that of wild-type cells with most biomass attaching close to the liquid-air interphase but also covering areas away from the liquid surface. Addition of Thanatos to more mature biofilms (after 22 h) also had a different effect on biofilm formation of both strains. While for the wild type addition of Thanatos slightly decreased the amount of surface-attached biomass, that of the mutant was significantly enhanced. Surprisingly, addition of heat-inactivated phage particles rather enhanced biofilm formation of both strains. In contrast to the biofilm formation behavior, the distinct negative effect of active Thanatos exposure to planktonic cells was highly similar for both strains (Supplementary Figure S7).

To further determine the effects of Thanatos exposure on older biofilms, *S. oneidensis* biofilms of both the wild-type and the prophage-lacking mutant strain were allowed to develop for 24 h in microtiter plates before Thanatos was added. The cultures were then incubated for additional 2, 6, 9, or 24 h before the amount of planktonic cells and the attached biomass were analyzed. Again, the values were compared to biofilm cultures to which no phages were added. For wild-type cells, a 2-h exposure to the phage slightly decreased surface-associated biomass (Figure 7B), and a potential slight enhancing effect was observed after 6 and 9 h exposure to Thanatos. However, after 24 h of exposure to Thanatos, surface-attached biomass was drastically decreased to >40% of that of untreated cultures. In contrast, the surface-associated biomass of the prophage-lacking mutants gradually decreased slightly with increased incubation (Figure 7D).

Overall, the data strongly suggests that the presence of Thanatos affects the amount and morphology of surface-associated biomass in a way that depends on the developmental biofilm stage and the presence or absence of the *S. oneidensis* prophages LambdaSo and MuSo2. The latter two phages have been shown to be required for normal biofilm formation of *S. oneidensis*, which is thought to be due to a release of biofilm-promoting factors, such as extracellular DNA, by phage-mediated lysis (Gödeke et al., 2011; Binnenkade et al., 2014). We therefore determined the occurrence of extracellular DNA in the presence or absence of phage Thanatos. To this end, biofilms of both strains in the presence or absence of phage Thanatos were developed and the cultures were then stained to visualize cells and extracellular DNA by microscopy (Figure 7E). As expected, in the absence of Thanatos more extracellular DNA occurred in wild-type cell cultures compared to those of the prophage mutant. In the presence of Thanatos, the amount of extracellular DNA and presumably dead cells was drastically increased for both strains. In addition, more densely packed cell aggregates were observed. Thus, exposure to Thanatos likely increases cell lysis and thus the release of potential biofilm-affecting factors such as extracellular DNA.

## Discussion

In this study, we have aimed at further exploring phages preying on species of the highly widespread genus *Shewanella*, and here we describe the isolation of two phages, *Shewanella* phages Thanatos-1 and Thanatos-2, infecting and lysing *S. oneidensis*. Both phages are closely related and constitute a novel group within the *Tevenvirinae*, and we have characterized Thanatos-1 in more detail. In agreement with the genome-based classification, Thanatos exhibits the morphology of the *Tevenvirinae* with a head, a contractile tail, collar and baseplate decorated with tail fibers. Under the conditions tested, Thanatos exhibited a strictly lytic life cycle with a rather small burst size of about 20 virions per cell after a latent period of about 40 min. Adsorption of phage Thanatos to *S. oneidensis* cells required the *waaC* gene product, which, in *E. coli*, is involved in formation of the outer membrane lipopolysaccharide (Grizot et al., 2006). This strongly suggests that phage Thanatos uses the LPS of *S. oneidensis* as the major primary attachment site, which is not uncommon among *Myoviridae* (Nobrega et al., 2018), and effective phage binding occurred within several minutes. Among other *Shewanella* sp. tested, Thanatos-induced lysis occurred with some other – but not all – *S. oneidensis*, as well as with two *S. seohaensis* isolates and *Shewanella* sp. MR-7. This is likely due to variations in the LPS structure of *Shewanella* strains (Korenevsky et al., 2002; Vinogradov et al., 2003, 2008; Korenevsky and Beveridge, 2007) or the absence of a crucial yet unknown secondary surface target. If the host range extends to bacterial species from other genera exhibiting a similar LPS structure remains to be shown.

With a dsDNA genome of about 161 kbp, Thanatos possesses the largest genome described for *Shewanella*-infecting phages so far. Less than half of the 206 gene products predicted to be encoded on the chromosome, namely 92, have an annotated function, mainly comprising proteins already identified in other phages (e.g., 66 in *E. coli* T4) and represent proteins involved in phage morphogenesis and its regulation, nucleotide metabolism and DNA packaging. Thus, at least 114 proteins are of yet unknown function, which is a common finding in phages with larger genomes. The majority of the phage proteins, 155, were identified in PEG-precipitated cultures of lysed cells, and among those were numerous hypothetical proteins, which do not occur in the purified phage particles. While the identification by MS does not allow proper quantification, the amount of several peptides identified suggested that some of these proteins of unknown function may occur at a rather high abundance. This may indicate a vital role in the propagation of phage Thanatos, which is currently under further investigation.

In recent years, phages have received increasing interest as a potential control agent against harmful or simply unwanted bacterial species in clinical, agricultural and industrial settings (Lemire et al., 2018; Kortright et al., 2019; Lewis and Hill, 2020). As some *Shewanella* species are implicated in food spoilage, in particular with respect to fish products, several studies have addressed potential applications of appropriate phages to prevent propagation or to target existing communities of *Shewanella* sp. (Han et al., 2014; Li et al., 2014; Zhang et al., 2018; Yang et al., 2019). However, in most environments the majority of bacteria does not exist as single planktonic cells, but as surface-associated complex communities, commonly referred to as biofilms, where the cells are embedded in a self-produced matrix (Flemming and Wuertz, 2019). Thus, phages typically do not face freely diffusing or swimming single cells, e.g., as in regular phage adhesion assays, but cells in biofilms. This significantly changes the way phages and host cells interact as matrix components may limit phage diffusion and host access. In addition, metabolically dormant cells, which frequently occur in biofilms, may not be competent for phage propagation, and furthermore, biofilm cells often activate phage defense systems (reviewed in Hansen et al., 2019). The properties of the matrix and the cells within depend on the developmental stage of the biofilm. Accordingly, different effects of phage exposure to biofilm cells can be expected. We therefore determined the effect of phage Thanatos on nascent and mature biofilms of *S. oneidensis*.

Phage particles added to existing biofilms of wild-type cells grown under static conditions lead to a significant reduction of both surface-associated biomass and planktonic cells if the incubation is sufficiently long, in our case 24 h. This indicates that, under the conditions tested, Thanatos has access to the cell surfaces to attach via the LPS of the cells and that the cells provide enough metabolic activity to allow successful phage propagation. Generally, the matrix of *S. oneidensis* biofilms is thought to consist of polysaccharides, proteins, DNA and likely also membrane vesicles (Thormann et al., 2004; Saville et al., 2010; Pirbadian et al., 2014), which may shield the cell surface from phage interaction. Accordingly, some phages have been demonstrated to carry proteins, which are able to degrade matrix components to allow the phages to reach the designated adhesion structures (Samson et al., 2013). So far, the genome analysis does not reveal any obvious candidates for such enzymes to be encoded in the Thanatos genome. However, such gene products may be present among the many predicted proteins of unknown function. In addition, the *S. oneidensis* biofilm morphology as well as the structure and composition of the matrix under the applied conditions is yet unknown and may provide sufficient room for the Thanatos particles to diffuse in. Notably, the effect of Thanatos-mediated degradation was less pronounced for existing biofilms of *S. oneidensis* mutants lacking the prophages LambdaSo and MuSo2. These biofilms are supposed to contain a lower amount of extracellular DNA (Gödeke et al., 2011) and may therefore exhibit a different biofilm morphology.

Also when Thanatos phage particles were added along with the planktonic cells at the beginning of inoculation, subsequent biofilm formation was significantly affected. We found that the surface-associated biomass of wild-type cells surpassed that of non-infected cultures by about a half, while biofilms of a LambdaSo/MuSo2 mutant display a different macroscopic morphology. A similar biofilm-promoting effect has been reported for the lytic SFCi1 phage infecting *Shewanella fidelis* 3313 (Leigh et al., 2017) and also for other systems as well, e.g., for *Staphylococcus aureus* (Hosseinidoust et al., 2013; Fernandez et al., 2017) and *Vibrio anguillarum* (Tan et al., 2015). The study on *S. fidelis* 3313 revealed an increased amount of extracellular DNA in the matrix of the developed biofilms when the cells were exposed to phages early on (Leigh et al., 2017). The authors propose that the release of DNA from phage-killed cells serves as an early structural component, which then enhances biofilm formation. This hypothesis was also discussed for the *Staphylococcus* system (Hosseinidoust et al., 2013). Our microscopic data strongly indicates an accumulation of dead cells and extracellular DNA upon exposure to Thanatos, thus, the release of this or other biofilm-affecting compounds through phage-mediated cell lysis may similarly be the major factor of the observed differences in *S. oneidensis* biofilm formation. Another possibility is that exposure to phages (or death and lysis of neighboring cells) can be sensed by bacteria, which in turn produce more extracellular substances to increase biofilm formation, or foster cell-cell interaction, as a means for phage defense. As an example, *S. aureus* was observed to induce the stringent response upon exposure to non-lethal doses of phage philPLA-RODI, which was proposed to slow down phage propagation through the community (Fernandez et al., 2017). In addition, selective pressure exerted by phage exposure may lead to the enrichment of phage-tolerant mutants with better abilities for surface and cell-to-cell interactions, which form more robust biofilms. In this respect, we also tested the biofilm formation of the *S. oneidensis* Δ*waaC* mutant, which was resistant to phage infection. However, no difference in biofilm formation compared to that of wild-type cells was observed (data not shown). Thus, the results implicate that the cellular response to phage exposure may not always yield the expected outcome and is probably highly dependent on the phage-host pair. The different effects phage Thanatos exerts on the biofilm formation of its host *S. oneidensis* and if the phage contains gene products that may be of general use to contain bacteria or for biofilm control is currently under investigation.

## Data Availability Statement

The datasets presented in this study can be found in online repositories. The names of the repository/repositories and accession number(s) can be found in the article/ Supplementary Material.

## Author Contributions

MK, AD, DB, NS, SB, JH, TG, AB, JK, and KT conceived the experiments. MK, AD, DB, NS, TL, FH, SB, JH, TG, and MR conducted the experiments. MK, AD, DB, NS, SB, JH, TG, AB, JK, and KT discussed the results. DB, JH, TG, NS, and KT wrote the manuscript. All authors contributed to the article and approved the submitted version.

## Conflict of Interest

The authors declare that the research was conducted in the absence of any commercial or financial relationships that could be construed as a potential conflict of interest.
