# Supplementary material for: Isolation and Characterization of Shewanella Phage Thanatos Infecting and Lysing Shewanella oneidensis and Promoting Nascent Biofilm Formation
Source: Front Microbiol. 2020 Sep 18;11:573260. doi: 10.3389/fmicb.2020.573260 (PMC7530303; doi:10.3389/fmicb.2020.573260)
Supplement: Supplementary file 1 [file Data_Sheet_1.pdf]

Supplementary Material

Supplemental Table 1: Bacterial strains and plasmids used in this study

Strain or plasmid	Relevant genotype or description	Source or reference
Escherichia coli		
DH5α λpir	*recA1 gyrA* (*lacIZYA-argF*) (80d *lac* [*lacZ*] M15) *pir* RK6, cloning strain	(Miller and Mekalanos, 1988)
WM3064	*thrB1004 pro thi rpsL hsdS lacZ* ΔM15 RP4-1360 Δ(*araBAD*) 567Δ*dapA* 1341::[*erm pir*(wt)], conjugation strain for *Shewanella*	W. Metcalf, University of Illinois, Urbana-Champaign
MG1655	K12 wild type	(Jensen, 1993)
Shewanella putrefaciens CN-32		
S798	Wild type	(Fredrickson et al., 1998)
S2652	Δ*cas1_2*	(Dwarakanath et al., 2015)
Shewanella oneidensis MR-1		
S79	Wild type	(Venkateswaran et al., 1999)
S6593	ΔLambdaSo ΔMuSo2	This work
S6100	ΔLambdaSo ΔMuSo2 Δ*waaC*	This work
S6118	Δ*waaC*	This work
S6160	Δ*waaC::waaC*	This work
S6161	ΔLambdaSo ΔMuSo2 Δ*waaC::waaC*	This work
Shewanella oneidensis	Isolate No. S17	(Jung-Schroers et al., 2017)
Shewanella oneidensis	Isolate No. S45	(Jung-Schroers et al., 2017)
Shewanella oneidensis	Isolate No. S52	(Jung-Schroers et al., 2017)
Shewanella oneidensis	Isolate No. S54	(Jung-Schroers et al., 2017)
Shewanella oneidensis	Isolate No. S62	(Jung-Schroers et al., 2017)
Shewanella oneidensis	Isolate No. S63	(Jung-Schroers et al., 2017)
Shewanella oneidensis	Isolate No. S66	(Jung-Schroers et al., 2017)
Shewanella oneidensis	Isolate No. S69	(Jung-Schroers et al., 2017)
Shewanella oneidensis	Isolate No. S74	(Jung-Schroers et al., 2017)
**Further *Shewanella* species**		
S. sp. MR-4	Wild type	(Nealson et al., 1991)
S. sp. MR-7	Wild type	(Nealson et al., 1991)
S. sp.	Isolate P1-19	M. Agler-Rosenbaum, Hans-Knöll Institute, Jena, Germany

Supplemental Table 1 – continued

S. sp.	Isolate P1-23	M. Agler-Rosenbaum, Hans-Knöll Institute, Jena, Germany
S. amazonensis SB2B	Wild type	(Venkateswaran et al., 1998)
S. sp. ANA-3	Wild type	(Saltikov et al., 2003)
S. sp. W3-18-1	Wild type	(Murray et al., 2001)
S. baltica OS195	Wild type	(Brettar et al., 2001)
S. baltica	Isolate No. S4	(Jung-Schroers et al., 2018)
S. baltica	Isolate No. S7	(Jung-Schroers et al., 2018)
S. baltica	Isolate No. S37	(Jung-Schroers et al., 2018)
S. baltica	Isolate No. S38	(Jung-Schroers et al., 2018)
S. baltica	Isolate No. S44	(Jung-Schroers et al., 2018)
S. baltica	Isolate No. S50	(Jung-Schroers et al., 2018)
S. seohaensis	Isolate No. S8	(Jung-Schroers et al., 2018)
S. seohaensis	Isolate No. S10	(Jung-Schroers et al., 2018)
S. seohaensis	Isolate No. S11	(Jung-Schroers et al., 2018)
S. seohaensis	Isolate No. S31	(Jung-Schroers et al., 2018)
S. seohaensis	Isolate No. S32	(Jung-Schroers et al., 2018)
**Other species**		
Pseudomonas putida KT2440	Wild type	(Nelson et al., 2002)
**Plasmids**		
pNPTS138-R6KT	*mob*RP4$^+$ *ori*-R6K *sacB*, suicide plasmid for in frame deletions or insertions in *S. putrefaciens* and *S. oneidensis*, Km^r	(Lassak et al., 2010)
pNPTS138-R6KT ΔLambdaSo	Lambda So prophage in frame deletion vector	(Gödeke et al., 2011)
pNPTS138-R6KT ΔMuSo2	MuSo2 prophage in frame deletion vector	(Gödeke et al., 2011)
pNPTS138-R6KT Δ*waaC*	*waaC* in frame deletion vector	This work
pNPTS138-R6KT KI *waaC*	*waaC* in frame insertion vector	This work
pMiniHimar RB1	Transposon mutagenesis vector	(Bouhenni et al., 2005)

Supplemental Table 2: Oligonucleotides used in this study

Identifier	Primer name	Sequence 5´→ 3´
**Plasposon sequencing**		
FR24	pMiniHimar fw	TCGGGTATCGCTCTTGAAGGG
FR25	pMiniHimar rv	CATTTAATACTAGCGACGCCATCT
***waaC* deletion or complementation**		
TL130	SO_waaC_KO_up_fw	GCCAAGCTTCTCTGCAGGATCCAAGTCTTCGGCACCATCG
TL131	SO_waaC_KO_up_rv	GGGTGACAGGCTAACAGTGGGCGAATAGCCCTTAAGGTTATTT
TL132	SO_waaC_KO_down_fw	TAACCTTAAGGGCTATTCGCCCACTGTTAGCCTGTCACCC
TL133	SO_waaC_KO_down_rv	GCGAATTCGTGGATCCAGATAATGCTGGCCATTAAGTGCC
TL134	Check_waaC_fw	CGATGAAACTGCGCAATGGTC
TL135	Check_waaC_rv	CGATGGCTATGGCTATCCGC

Supplemental Table 3: Thanatos gene annotation and protein presence in phage lysates

Gene	putative function	Identified by MS in purified phages	PEG precipitate
TH1_001	Phage terminase, small subunit	X	
TH1_002	Phage terminase, large subunit	X	
TH1_003	Phage tail sheath protein	X	X
TH1_004	Phage tail tube protein	X	X
TH1_005	Phage portal (connector) protein	X	X
TH1_006	hypothetical protein		X
TH1_007	Phage capsid and scaffold	X	X
TH1_008	Phage prohead assembly (scaffolding) protein	X	X
TH1_009	Phage prohead assembly (scaffolding) protein	X	X
TH1_010	Phage major capsid protein	X	X
TH1_011	Phage capsid vertex	X	X
TH1_012	hypothetical protein		X
TH1_013	Phage head decoration protein	X	X
TH1_014	RNA ligase	X	X
TH1_015	hypothetical protein		X
TH1_016	Phage inhibitor of prohead protease	X	X
TH1_017	DNA helicase	X	X
TH1_018	hypothetical protein		
TH1_019	Single stranded DNA-binding protein		X
TH1_020	Phage baseplate wedge subunit / Phage tail lysozyme	X	X
TH1_021	Phage baseplate hub assembly chaperone	X	X
TH1_022	Phage baseplate		
TH1_023	Phage baseplate hub subunit	X	X
TH1_024	Phage baseplate hub distal subunit		
TH1_025	Phage tail tape measure protein	X	X
TH1_026	Phage baseplate tail tube cap	X	X
TH1_027	Phage baseplate tail tube initiator	X	X
TH1_028	RNA polymerase-ADP-ribosyltransferase	X	X
TH1_029	RNA polymerase-ADP-ribosyltransferase	X	X
TH1_030	hypothetical protein		
TH1_031	Phage tail connector protein	X	X
TH1_032	Phage tail fiber protein (long tail fiber)	X	X
TH1_033	Phage ribonuclease H		X
TH1_034	Double-stranded DNA binding protein		
TH1_035	Phage late-transcription coactivator		

TH1_036	Phage DNA helicase loader		
TH1_037	Single-stranded DNA-binding protein	X	X
TH1_038	hypothetical protein		
TH1_039	Phage lysozyme		X
TH1_040	Phage endonuclease		X
TH1_041	Phage endoribonuclease		X
TH1_042	hypothetical protein		X
TH1_043	hypothetical protein	X	X
TH1_044	hypothetical protein	X	X
TH1_045	hypothetical protein		
TH1_046	nucleoid disruption protein	X	X
TH1_047	DNA topoisomerase II	X	X
TH1_048	Phage recombination-related endonuclease	X	X
TH1_049	Phage recombination-related endonuclease	X	X
TH1_050	hypothetical protein	X	X
TH1_051	Sliding clamp DNA polymerase accessory protein	X	X
TH1_052	Phage DNA polymerase clamp loader subunit		X
TH1_053	Phage DNA polymerase clamp loader subunit		
TH1_054	Phage endoribonuclease		X
TH1_055	hypothetical protein		
TH1_056	Phage DNA polymerase	X	X
TH1_057	hypothetical protein	X	X
TH1_058	hypothetical protein	X	X
TH1_059	hypothetical protein	X	X
TH1_060	hypothetical protein	X	X
TH1_061	hypothetical protein	X	X
TH1_062	Deoxycytidylate 5-hydroxymethyltransferase	X	X
TH1_063	hypothetical protein	X	X
TH1_064	hypothetical protein	X	X
TH1_065	hypothetical protein	X	X
TH1_066	hypothetical protein		
TH1_067	Phage recombination protein		X
TH1_068	Phage homing endonuclease		
TH1_069	hypothetical protein		
TH1_070	Phage recombination protein		X
TH1_071	Phage head assembly protein		
TH1_072	DNA helicase		X
TH1_073	hypothetical protein		
TH1_074	DNA primase		

TH1_075	dCTP pyrophosphatase		X
TH1_076	RNA ligase	X	X
TH1_077	hypothetical protein		X
TH1_078	hypothetical protein		X
TH1_079	hypothetical protein		X
TH1_080	hypothetical protein		
TH1_081	hypothetical protein		
TH1_082	hypothetical protein	X	X
TH1_083	hypothetical protein	X	
TH1_084	hypothetical protein		X
TH1_085	hypothetical protein	X	X
TH1_086	hypothetical protein		X
TH1_087	hypothetical protein		X
TH1_088	hypothetical protein		X
TH1_089	hypothetical protein		
TH1_090	hypothetical protein		
TH1_091	hypothetical protein		X
TH1_092	hypothetical protein		X
TH1_093	DNA helicase	X	X
TH1_094	hypothetical protein	X	X
TH1_095	hypothetical protein		X
TH1_096	Exonuclease		X
TH1_097	hypothetical protein	X	X
TH1_098	hypothetical protein	X	X
TH1_101	hypothetical protein		X
TH1_102	Dihydrofolat reductase		
TH1_103	Thymidylate synthase	X	X
TH1_104	Ribonucleotide reductase of class Ia (aerobic), alpha subunit	X	X
TH1_105	hypothetical protein		X
TH1_106	Ribonucleotide reductase of class Ia (aerobic), beta subunit	X	X
TH1_107	Endonuclease		
TH1_108	hypothetical protein		X
TH1_109	DNA topoisomerase large subunit	X	X
TH1_110	hypothetical protein		
TH1_111	hypothetical protein		X
TH1_112	DNA topoisomerase large subunit		X
TH1_113	hypothetical protein	X	X
TH1_114	hypothetical protein	X	X
TH1_115	hypothetical protein	X	X
TH1_116	hypothetical protein		X

TH1_117	hypothetical protein	X	X
TH1_118	Polynucleotide kinase	X	X
TH1_119	dCMP deaminase		X
TH1_120	Phage head assembly chaperone protein	X	X
TH1_121	hypothetical protein		
TH1_122	hypothetical protein	X	X
TH1_123	DNA ligase	X	X
TH1_124	hypothetical protein	X	X
TH1_125	hypothetical protein		
TH1_126	DNA adenine methylase		X
TH1_127	hypothetical protein		
TH1_128	Phage lysis inhibitor	X	X
TH1_129	Phage lysis inhibitor	X	X
TH1_130	hypothetical protein	X	X
TH1_131	hypothetical protein		X
TH1_132	hypothetical protein		X
TH1_133	hypothetical protein		
TH1_134	hypothetical protein		X
TH1_135	hypothetical protein		
TH1_136	hypothetical protein		X
TH1_137	hypothetical protein		X
TH1_138	hypothetical protein		X
TH1_139	hypothetical protein		X
TH1_140	hypothetical protein		X
TH1_141	hypothetical protein		X
TH1_142	hypothetical protein	X	X
TH1_143	hypothetical protein	X	X
TH1_144	hypothetical protein		X
TH1_145	hypothetical protein	X	X
TH1_146	hypothetical protein	X	X
TH1_147	hypothetical protein		X
TH1_148	hypothetical protein		X
TH1_149	hypothetical protein		
TH1_150	hypothetical protein	X	X
TH1_151	hypothetical protein		X
TH1_152	hypothetical protein		X
TH1_153	hypothetical protein	X	X
TH1_154	hypothetical protein		
TH1_155	hypothetical protein		X
TH1_156	hypothetical protein		
TH1_157	hypothetical protein		
TH1_158	hypothetical protein		X
TH1_159	hypothetical protein		X

TH1_160	hypothetical protein	X	X
TH1_161	hypothetical protein		X
TH1_162	hypothetical protein		X
TH1_163	hypothetical protein		X
TH1_164	hypothetical protein		X
TH1_165	hypothetical protein		X
TH1_166	hypothetical protein		X
TH1_167	Phage transcriptional regulator of middle promoters		
TH1_168	hypothetical protein		
TH1_169	t holin lysis mediator	X	X
TH1_170	Phage tail fibers	X	X
TH1_171	Phage tail connector protein	X	X
TH1_172	hypothetical protein	X	
TH1_173	hypothetical protein	X	X
TH1_174	hypothetical protein		
TH1_175	Phage sigma factor		X
TH1_176	hypothetical protein		
TH1_177	hypothetical protein		
TH1_178	Phage endonuclease		
TH1_179	hypothetical protein		
TH1_180	Thioredoxin		X
TH1_181	hypothetical protein	X	X
TH1_182	T4-like phage protein	X	X
TH1_183	hypothetical protein	X	X
TH1_184	hypothetical protein		
TH1_185	hypothetical protein	X	X
TH1_186	hypothetical protein	X	X
TH1_187	hypothetical protein	X	X
TH1_188	putative deoxynucleoside monophosphate kinase	X	X
TH1_189	Phage tail completion protein	X	X
TH1_190	DNA end protector protein	X	X
TH1_191	Phage head completion protein		
TH1_192	Phage baseplate wedge subunit	X	X
TH1_193	Phage baseplate hub subunit / Phage tail lysozyme	X	X
TH1_194	hypothetical protein	X	X
TH1_195	hypothetical protein	X	X
TH1_196	Phage baseplate wedge subunit	X	X
TH1_197	hypothetical protein		X
TH1_198	Phage baseplate wedge initiator	X	X

TH1_199	Phage baseplate wedge subunit	X	X
TH1_200	hypothetical protein		
TH1_201	Phage baseplate wedge tail fiber connector	X	X
TH1_202	Phage baseplate wedge subunit and tail pin	X	X
TH1_203	Phage baseplate wedge subunit	X	X
TH1_204	Phage straight tail fiber (short tail fiber)	X	X
TH1_205	Phage neck whiskers	X	X
TH1_206	Phage head completion, neck hetero-dimeric protein	X	X
TH1_207	Phage head completion, neck hetero-dimeric protein	X	X
TH1_208	Phage tail completion protein	X	X

Supplemental Table 4: Determination of the host range of Thanatos. Table visualizes the result of a phage spot assay performed on different host strains to determine the host range of Thanatos.

Strain	relevant genotype and/or description	Thanatos-mediated plaque formation
S. oneidensis	MR-1	Yes
S. oneidensis	MR-1 ΔLambdaSo ΔMuSo2	Yes
S. oneidensis	Isolate No. S17	No
S. oneidensis	Isolate No. S52	No
S. oneidensis	Isolate No. S54	No
S. oneidensis	Isolate No. S62	Yes
S. oneidensis	Isolate No. S63	Yes
S. oneidensis	Isolate No. S66	Yes
S. oneidensis	Isolate No. S69	No
S. oneidensis	Isolate No. S74	Yes
S. baltica	OS195	No
S. baltica	Isolate No. S4	No
S. baltica	Isolate No. S7	No
S. baltica	Isolate No. S37	No
S. baltica	Isolate No. S38	No
S. baltica	Isolate No. S44	No
S. baltica	Isolate No. S50	No
S. seohaensis	Isolate No. S8	Yes
S. seohaensis	Isolate No. S10	No
S. seohaensis	Isolate No. S11	Yes
S. seohaensis	Isolate No. S31	No
S. seohaensis	Isolate No. S32	No
S. amazonensis	SB2B	No
S. putrefaciens	CN-32	No
S. putrefaciens	CN-32 Δcas1_2	No
S. sp.	Isolate P1-23	No
S. sp.	Isolate P1-19	No
S. sp.	ANA-3	No
S. sp.	W3-18-1	No
S. sp.	MR-4	No
S. sp.	MR-7	Yes
P. putida	KT2440	No
E. coli	MG 1655	No

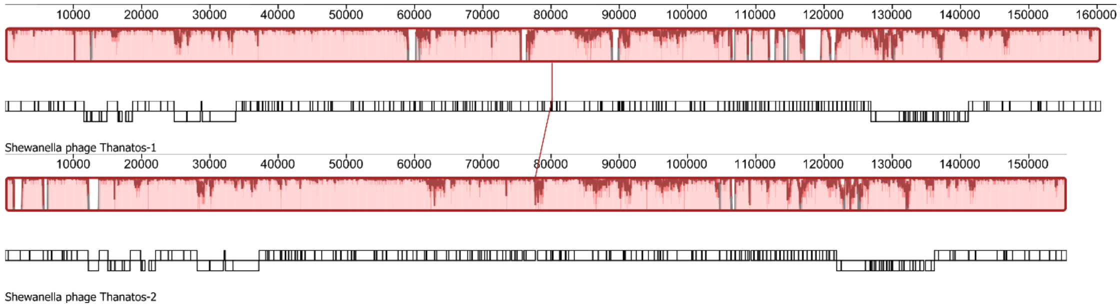

Supplemental Figure 1: Whole-genome alignment of Thanatos-1 and Thanatos-2 created using mauve within Geneious prime 2020. Both genomes share 87.8 % pairwise identity as determined using the Geneious aligner, which employs a progressive pairwise alignment algorithm similar to ClustalW.

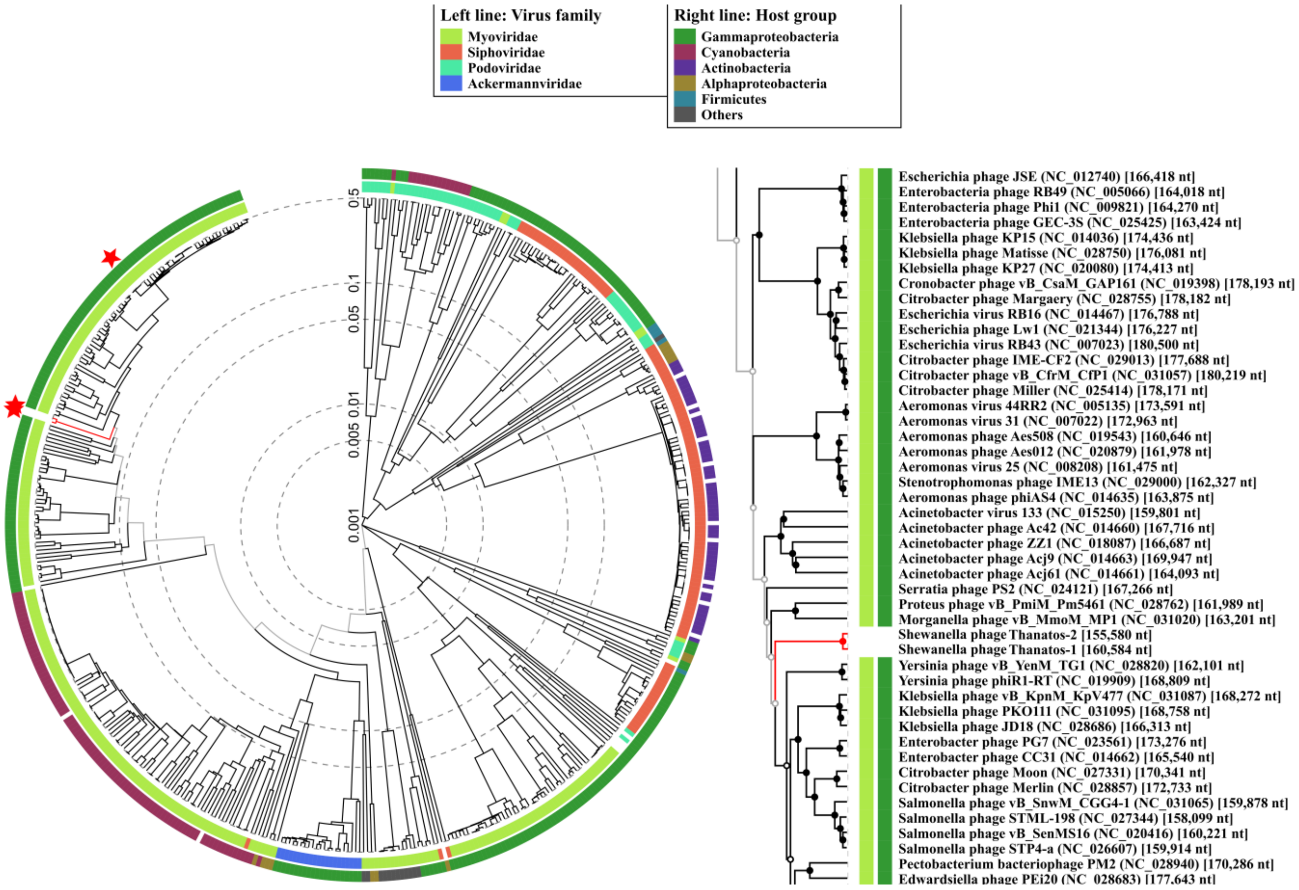

Supplemental Figure 2: Thanatos-1 and Thanatos-2 belong to the Myoviridae family according to viral proteomic tree. Viral proteomic tree analyses of Thanatos-1 and Thanatos-2, based on genome-wide sequence similarities computed by tBLASTx. Circular tree (left) shows all related viral genomes included in the ViPTree database with Thanatos phages and Escherichia virus T4 highlighted by red stars. Rectangular tree on the right shows the detailed phylogenetic tree of phage genomes in direct vicinity of Thanatos phages.

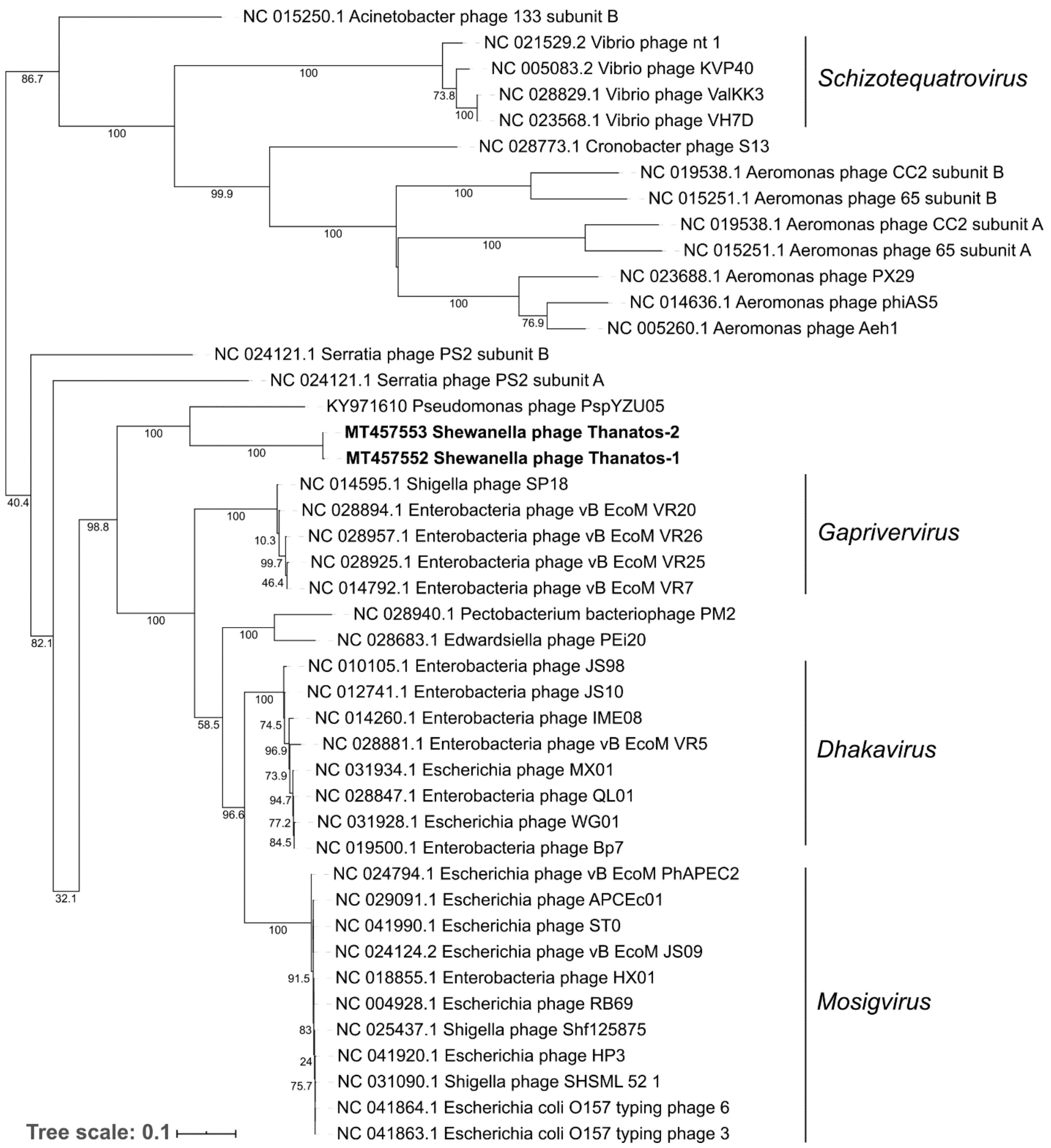

Supplemental Figure 3: Phylogeny based on DNA polymerase protein sequences. Phylogenetic trees were calculated using the Maximum-Likelihood algorithm with IQ-Tree (Trifinopoulos et al., 2016) employing the WAG substitution model (1000 bootstrap replicates) from multiple amino acid sequence alignments, which were created with Clustal Omega of DNA Polymerase including homologous proteins from the Tevenvirinae family. Visualization was created using Interactive Tree of Life (iTOL) (Letunic and Bork, 2007).

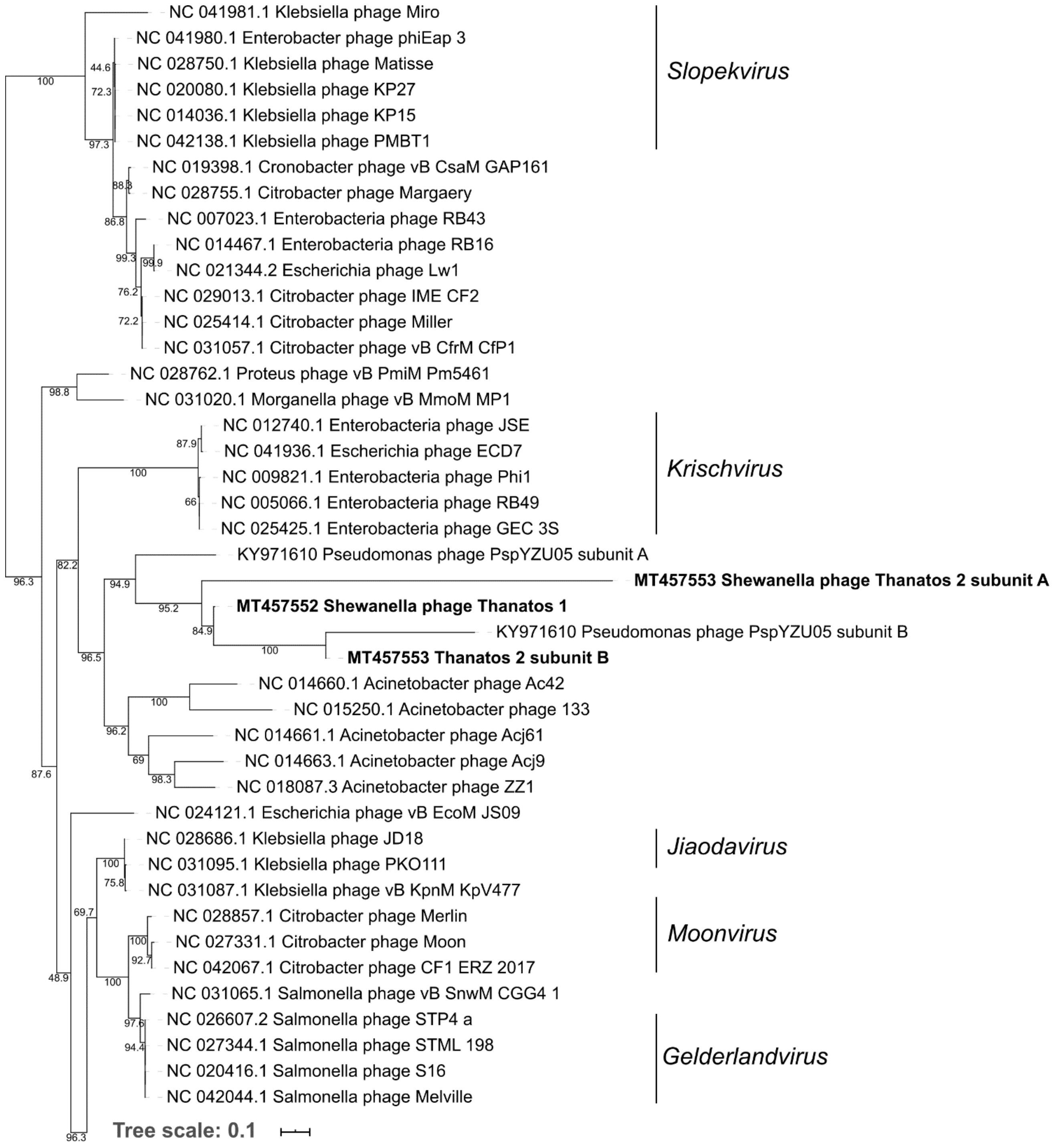

Supplemental Figure 4: Phylogeny based on terminase large subunit protein sequences. Phylogenetic trees were calculated using the Maximum-Likelihood algorithm with IQ-Tree (Trifinopoulos et al., 2016) employing the WAG substitution model (1000 bootstrap replicates) from multiple amino acid sequence alignments, which were created with Clustal Omega of large terminase subunit including homologous proteins from the Tevenvirinae family.Visualization was created using Interactive Tree of Life (iTOL) (Letunic and Bork, 2007).

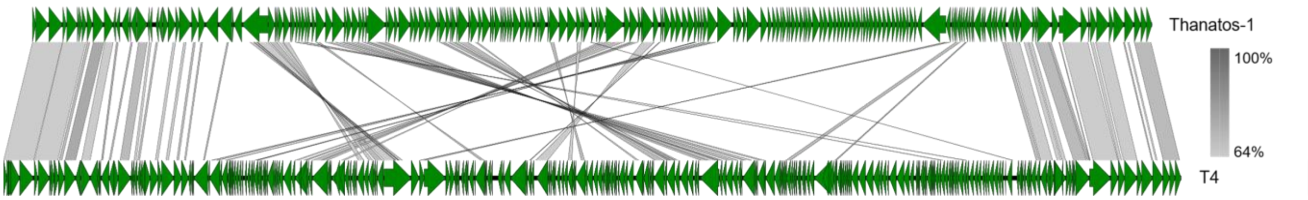

Supplemental Figure 5: Whole-genome comparison of *Shewanella* phage Thanatos-1 and *Escherichia* virus T4 created using easyfig (Sullivan, 2011). Similarities are based on tBLASTx comparison of both genomes.

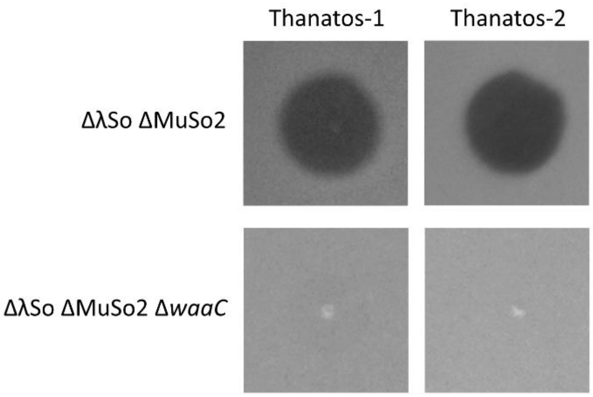

Figure 6. Deletion of *waaC* protects *Shewanella oneidensis* MR-1 from Thanatos-1 as well as from Thanatos-2 infection. Spot Assays of Thanatos-1 and Thanatos-2 on *Shewanella oneidensis* MR-1 ΔLambdaSo ΔMuSo2 and *Shewanella oneidensis* MR-1 ΔLambdaSo ΔMuSo2 Δ*waaC*.

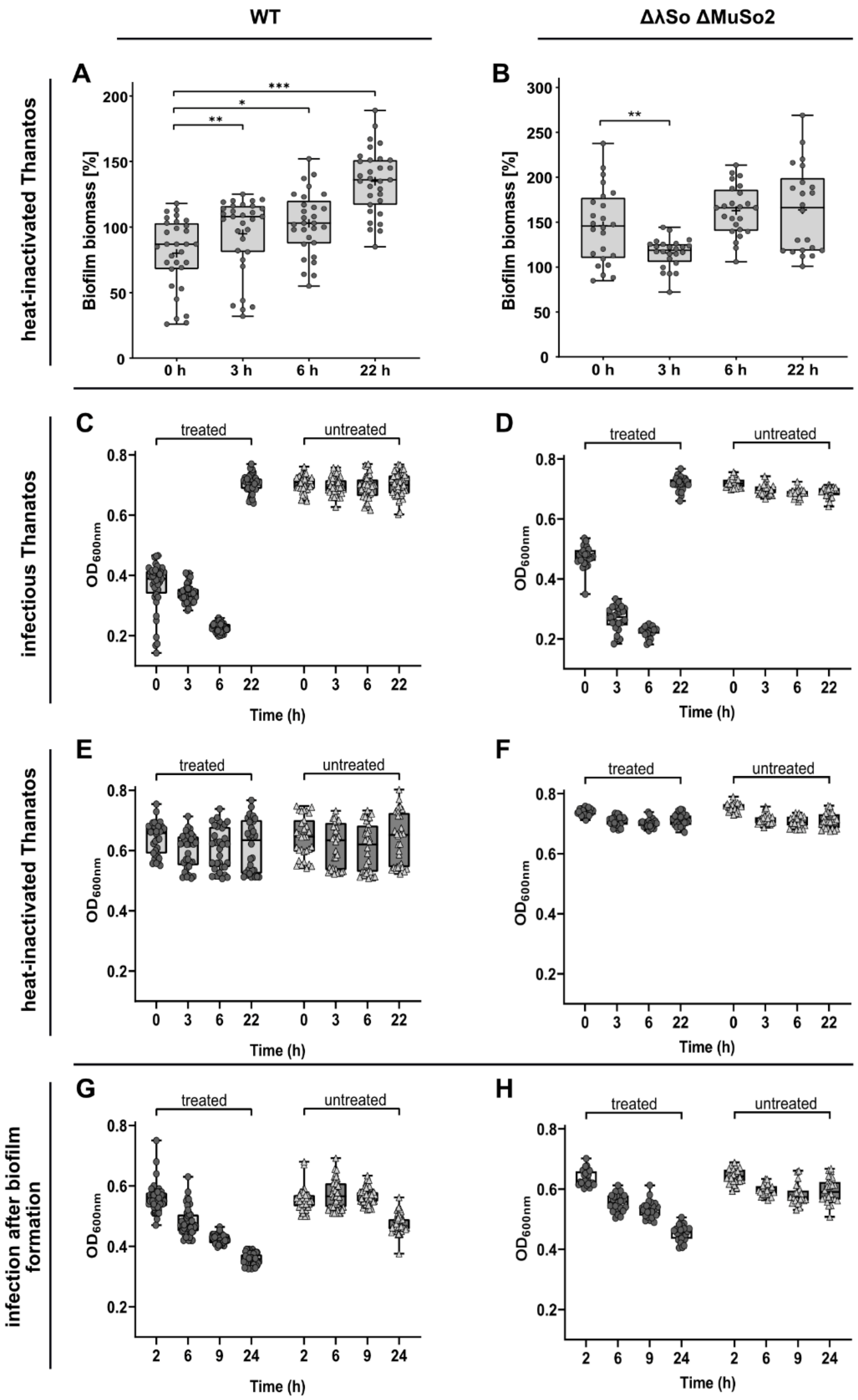

Supplemental Figure 7. Heat-inactivated static biofilm assays and optical densities of al static biofilm assays. For the static biofilm assays (Fig. 6), biofilm formation was calculated as the percentage of treated cultures (addition of either infectious or inactivated Thanatos) compared to untreated cultures (addition of an equal volume of LB medium), without normalization to the (planktonic) optical density. **(A + B)** Static biofilm assays with heat-inactivated Thanatos. **(C-F)** Optical density of short term biofilm formation assay. Phage (treated) or LB (untreated) was added to the nascent biofilm. **(G + H)** Optical density of long term biofilm assay. Phage (treated) or LB (untreated) was added to the mature biofilm.

Additional References

Bouhenni, R., Gehrke, A., and Saffarini, D. (2005). Identification of genes involved in cytochrome c biogenesis in *Shewanella oneidensis*, using a modified mariner transposon. Appl. Environ. Microbiol. 71(8)**,** 4935-4937. doi: 10.1128/AEM.71.8.4935-4937.2005.

Brettar, I., Moore, E.R., and Höfle, M.G. (2001). Phylogeny and abundance of novel denitrifying bacteria isolated from the water column of the central Baltic Sea. Microb. Ecol. 42(3)**,** 295-305. doi: 10.1007/s00248-001-0011-2.

Dwarakanath, S., Brenzinger, S., Gleditzsch, D., Plagens, A., Klingl, A., Thormann, K., et al. (2015). Interference activity of a minimal Type I CRISPR-Cas system from *Shewanella putrefaciens*. Nucleic Acids Res. 43(18)**,** 8913-8923. doi: 10.1093/nar/gkv882.

Fredrickson, J.K., Zachara, J.M., Kennedy, D.V., Dong, H., Onstott, T.C., Hinman, N.W., et al. (1998). Biogenic iron mineralization accompanying the dissimilatory reduction of hydrous ferric oxide by a groundwater bacterium. Geochim. Cosmochim. Acta 62**,** 3239-3257.

Gödeke, J., Paul, K., Lassak, J., and Thormann, K.M. (2011). Phage-induced lysis enhances biofilm formation in *Shewanella oneidensis* MR-1. *ISME J* 5(4)**,** 613-626. doi: ismej2010153 [pii] 10.1038/ismej.2010.153.

Jensen, K.F. (1993). The *Escherichia coli* K-12 "wild types" W3110 and MG1655 have an *rph* frameshift mutation that leads to pyrimidine starvation due to low *pyrE* expression levels. J. Bacteriol. 175(11)**,** 3401-3407. doi: 10.1128/jb.175.11.3401-3407.1993.

Jung-Schroers, V., Jung, A., Ryll, M., Bauer, J., Teitge, F., Steinhagen, D. (2018). Methods for identification and differentiation of different *Shewanella* spp. isolates for diagnostic use. J. Fish Dis. 41(4), 689-714. doi:10.1111/jfd.12772

Lassak, J., Henche, A.L., Binnenkade, L., and Thormann, K.M. (2010). ArcS, the cognate sensor kinase in an atypical Arc system of *Shewanella oneidensis* MR-1. Appl. Environ. Microbiol. 76(10)**,** 3263-3274. doi: AEM.00512-10 [pii] 10.1128/AEM.00512-10.

Letunic, I., and Bork, P. (2007). Interactive Tree Of Life (iTOL): an online tool for phylogenetic tree display and annotation. Bioinformatics 23(1)**,** 127-128. doi: btl529 [pii] 10.1093/bioinformatics/btl529.

Miller, V.L., and Mekalanos, J.J. (1988). A novel suicide vector and its use in construction of insertion mutations: osmoregulation of outer membrane proteins and virulence determinants in *Vibrio cholerae* requires *toxR*. J. Bacteriol. 170(6)**,** 2575 - 2583.

Murray, A.E., Lies, D., Li, G., Nealson, K., Zhou, J., and Tiedje, J.M. (2001). DNA/DNA hybridization to microarrays reveals gene-specific differences between closely related microbial genomes. Proc. Natl. Acad. Sci. U. S. A. 98(17)**,** 9853-9858.

Nealson, K.H., Myers, C.R., and Wimpee, B.B. (1991). Isolation and identification of manganese-reducing bacteria and estimates of microbial Mn(IV)-reducing potential in the Black Sea. Deep Sea Re.s Part A. Oceanograph Res. Papers 38**,** 907-920.

Nelson, K.E., Weinel, C., Paulsen, I.T., Dodson, R.J., Hilbert, H., Martins dos Santos, V.A., et al. (2002). Complete genome sequence and comparative analysis of the metabolically versatile *Pseudomonas putida* KT2440. Environ. Microbiol. 4(12)**,** 799-808. doi: 10.1046/j.1462-2920.2002.00366.x.

Saltikov, C.W., Cifuentes, A., Venkateswaran, K., and Newman, D.K. (2003). The *ars* detoxification system is advantageous but not required for As(V) respiration by the genetically tractable *Shewanella* species strain ANA-3. Appl. Environ. Microbiol. 69(5)**,** 2800-2809.

Sullivan, M.J., Petty N.K., and Beatson S.A. (2011). Easyfig: a genome comparison visualizer. Bioinformatics. 27(7),1009-1010.

Trifinopoulos, J., Nguyen, L.T., von Haeseler, A., Minh, B.Q. (2016) W-IQ-TREE: a fast online phylogenetic tool for maximum likelihood analysis. Nucleic Acids Res. 44, W232-W235

Venkateswaran., K., Dollhopf, M.E., Aller, R., Stackebrandt, E., and Nealson, K.H. (1998). *Shewanella amazonensis* sp. nov., a novel metal-reducing facultative anaerobe from Amazonian shelf muds. Internat. J. Syst. Bacteriol. 3**,** 965-972.

Venkateswaran, K., Moser, D.P., Dollhopf, M.E., Lies, D.P., Saffarini, D.A., MacGregor, B.J., et al. (1999). Polyphasic taxonomy of the genus *Shewanella* and description of *Shewanella oneidensis* sp. nov. Internat. J. Syst. Bacteriol. 2**,** 705-724.
